# A three-gene expression signature predicts lymph node metastasis in cervical squamous cell carcinoma: development and validation using TCGA and clinical cohorts

**DOI:** 10.3389/fmed.2026.1797844

**Published:** 2026-05-13

**Authors:** Yachen Duan, Jiajia Li, Xiaohua Wu

**Affiliations:** Department of Gynecological Oncology, Fudan University Shanghai Cancer Center, Shanghai, China

**Keywords:** 3-gene expression signature, cervical squamous cell carcinoma, decision curve analysis, lymph node metastasis prediction, TCGA database, TRIPOD

## Abstract

**Background:**

Lymph node metastasis (LNM) is a major prognostic determinant in early-stage cervical squamous cell carcinoma (SCC); however, conventional preoperative imaging has demonstrated sensitivities below 60% for sub-centimeter metastases, resulting in treatment misallocation for approximately 20–30% of patients.

**Purpose:**

This study aimed to develop and validate a minimal gene expression signature that can be performed on routine biopsy specimens to predict preoperative LNM in cervical SCC.

**Methods:**

This retrospective biomarker discovery and validation study had a two-phase design. In the discovery phase, we analyzed transcriptomic data from 116 The Cancer Genome Atlas (TCGA) cervical SCC samples and identified differentially expressed genes (DEGs) using Benjamini–Hochberg (BH) false discovery rate correction (FDR < 0.10). Then, we refined them using the least absolute shrinkage and selection operator (LASSO) regression analysis and multivariate logistic regression analysis. The locked signature was independently validated in a prospectively collected cohort of 202 patients (101 LNM-positive patients and 101 LNM-negative patients) from the Fudan University Shanghai Cancer Center using quantitative reverse transcription–polymerase chain reaction (qRT-PCR), with histopathological lymphadenectomy confirmation as the reference standard. Performance was assessed based on area under the curve (AUC), sensitivity, specificity, predictive values, and bootstrap internal validation. Decision curve analysis and a combined molecular-clinical model were also evaluated.

**Results:**

Of the 231 DE genes, a three-gene signature (LOC494141, GLOD5, and GML) was identified by sequential filtering. In the independent validation cohort, the signature achieved an AUC of 0.745 (95% CI: 0.676–0.814), with a sensitivity of 62.38%, a specificity of 64.36%, a positive predictive value of 63.64%, and a negative predictive value of 63.11%. Bootstrap validation confirmed model robustness (optimism-corrected AUC: 0.722; calibration slope: 0.913; Hosmer–Lemeshow *p* = 0.387). A combined model integrating the signature with tumor size and lymphovascular space invasion achieved an AUC of 0.789 (95% CI: 0.724–0.854), with a significant incremental value [net reclassification improvement (NRI) = 0.42, *p* = 0.001; integrated discrimination improvement (IDI) = 0.065, *p* < 0.001]. Decision curve analysis demonstrated net clinical benefit across threshold probabilities of 20–70%. At a 20% population prevalence, the adjusted negative predictive value reached 87.3%.

**Conclusion:**

This three-gene expression signature provides clinically informative preoperative risk stratification for LNM in cervical SCC. Intended as a complementary tool within integrated clinical assessment frameworks rather than a standalone diagnostic tool, this affordable qRT-PCR-based assay holds particular promise for resource-limited settings, pending prospective multicenter validation.

## Introduction

Cervical cancer remains a significant global health burden, and squamous cell carcinoma (SCC) represents approximately 70–80% of cases (1, 2). Despite advances in screening and treatment, accurate risk stratification remains challenging, particularly regarding lymph node involvement, which is the single most important prognostic factor affecting both survival and treatment selection in early-stage disease (1).

The presence of lymph node metastasis (LNM) fundamentally dichotomizes treatment pathways for early-stage cervical cancer. Node-negative patients achieve excellent outcomes with surgical intervention alone, maintaining fertility potential and avoiding radiation-associated morbidity (3). Conversely, node-positive disease necessitates concurrent chemoradiotherapy, rendering initial surgical approaches not only unnecessary but potentially harmful because of combined modality toxicities (3, 4). This therapeutic divergence underscores the critical need for accurate preoperative LNM assessment.

Current diagnostic modalities have substantial limitations in detecting nodal involvement. Conventional imaging, including computed tomography and magnetic resonance imaging, exhibits sensitivities of below 60% for sub-centimeter metastases (5, 6). Although 18F-fluorodeoxyglucose positron emission tomography (PET) improves detection rates, false-negative rates approaching 13% remain clinically unacceptable for treatment planning (7, 8). Moreover, surgical staging via lymphadenectomy, although definitive, subjects all patients to operative risks and cannot guide initial treatment selection (9). These diagnostic inadequacies result in treatment misallocation for approximately 20–30% of patients, highlighting an urgent unmet clinical need.

Molecular biomarkers offer a promising approach for improving preoperative risk assessment. The Cancer Genome Atlas (TCGA) has revolutionized cancer biomarker discovery through comprehensive molecular profiling (10). The recent availability of cervical cancer transcriptomic data provides an unprecedented opportunity to identify LNM-associated gene expression signatures that could complement current staging modalities.

In this study, we report the development and validation of a gene expression signature for predicting lymph node metastasis in cervical SCC. Using The Cancer Genome Atlas (TCGA) RNA sequencing data as a discovery platform, we identified candidate genes associated with nodal involvement and subsequently validated their predictive performance in an independent clinical cohort. Our findings establish a clinically applicable three-gene signature that could enhance preoperative risk stratification and serve as a complementary tool in personalized treatment selection frameworks.

## Methods

### Study design and patient recruitment

The discovery phase used publicly available transcriptomic data from The Cancer Genome Atlas (TCGA) data portal.^1^ Level 3 RNA-seq expression data from 116 primary cervical squamous cell carcinoma samples were obtained from the Illumina HiSeq 2000 RNA sequencing platform, encompassing the expression profiles of 20,530 genes. Expression data were obtained as RNA-Seq by Expectation Maximization (RSEM)-normalized count estimates, which constitute the standard normalization pipeline used by the TCGA consortium, incorporating both within-sample transcript length normalization and between-sample upper-quartile normalization. Clinical information was retrieved from the Clinical Data section using Biospecimen Core Resource (BCR) identification numbers to link molecular and clinical data. Patient identifiers were anonymized prior to analysis to maintain confidentiality. All TCGA samples were treatment-naïve primary tumors collected prior to any therapeutic intervention, ensuring the integrity of the molecular signatures. As all TCGA cervical SCC samples in this analysis were processed on the Illumina HiSeq 2000 platform under standardized TCGA protocols, inter-batch technical variability was minimized. However, we acknowledge that formal batch effect assessment using principal component analysis or surrogate variable analysis was not performed, and this is recognized as a limitation of the discovery phase.

**Figure 1:**
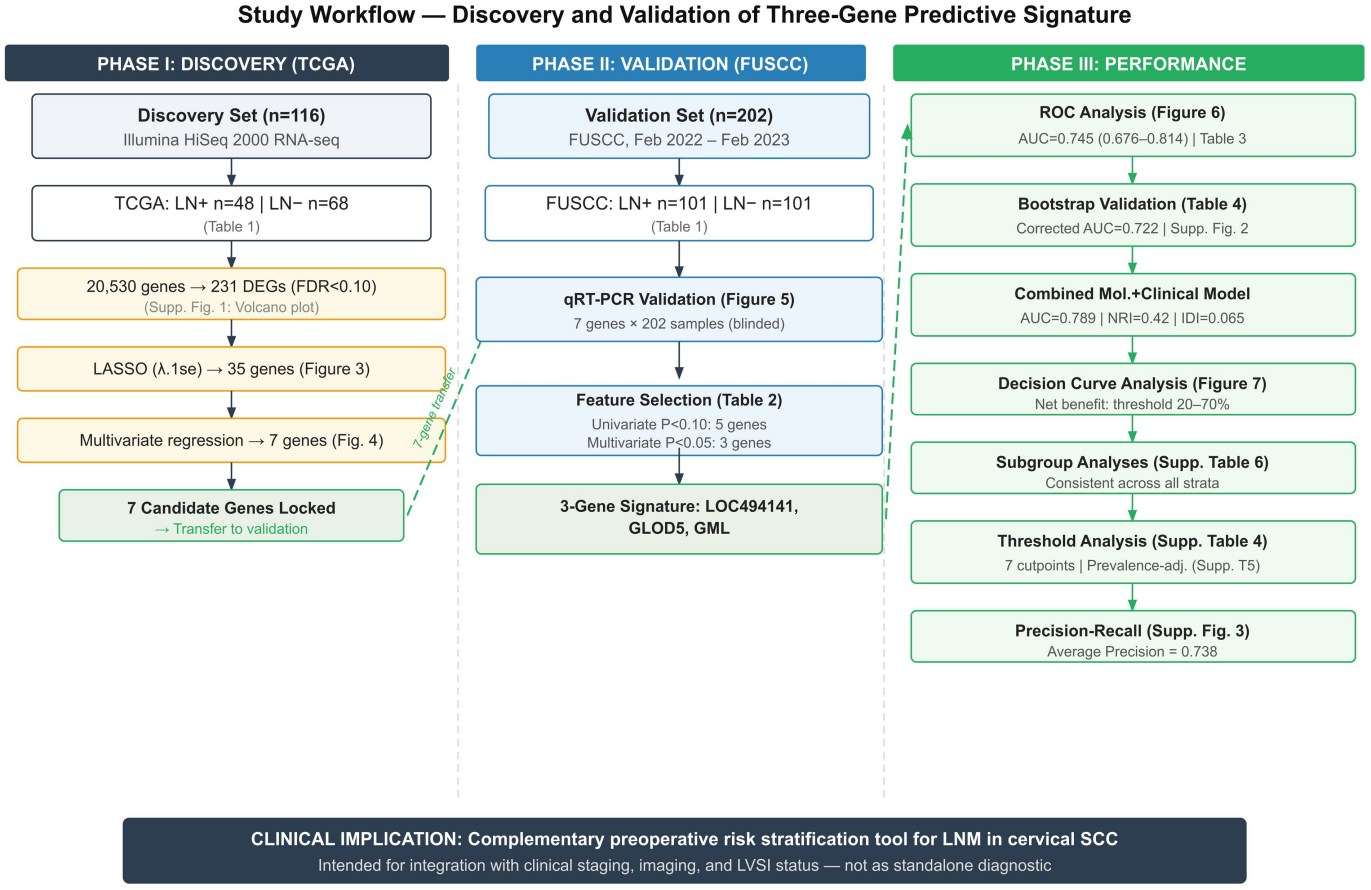
Study workflow diagram showing the three-phase design. Phase I (Discovery): 20,530 TCGA genes → 231 DEGs (FDR < 0.10; Supplementary Figure 1) → 35 LASSO-selected genes (lambda.1se; Figure 3) → 7 genes by multivariate regression (AUC = 0.938; Figure 4). Phase II (Validation): 7 genes validated by qRT-PCR in 202 FUSCC patients (Figure 5), yielding a 3-gene signature (Table 2). Phase III (Performance): AUC = 0.745 (Figure 6), bootstrap-corrected AUC = 0.722 (Table 4; Supplementary Figure 2), combined model AUC = 0.789, decision curve analysis confirming net benefit at 20–70% thresholds (Figure 7), consistent subgroup performance (Supplementary Table 6).

The independent validation cohort comprised 202 patients from the Fudan University Shanghai Cancer Center (FUSCC), with an equal representation of lymph node metastasis-positive (n = 101) and metastasis-negative (n = 101) cases to ensure balanced statistical power (Standards for Reporting of Diagnostic Accuracy Studies [STARD] flow diagram; Figure 1). This manuscript is reported in accordance with the guidelines of the Transparent Reporting of a Multivariable Prediction Model for Individual Prognosis or Diagnosis (TRIPOD) (11) and the Standards for Reporting of Diagnostic Accuracy Studies (STARD) (12); a completed TRIPOD and STARD checklist is provided in Supplementary Documents 1, 2. A 1:1 case–control design was selected to maximize statistical power for biomarker evaluation. However, we acknowledge that this artificial balancing does not reflect the true prevalence of lymph node metastasis in the source population (estimated at 15–25% for early-stage cervical SCC), and prevalence-dependent metrics (PPV, NPV) must be interpreted accordingly (Supplementary Table 5). Patients with histologically confirmed cervical squamous cell carcinoma were consecutively enrolled between February 2022 and February 2023. The diagnosis was confirmed by two independent, experienced pathologists at FUSCC, necessitating concordance study inclusion. Fresh tumor tissues were obtained during surgical resection and immediately flash frozen in liquid nitrogen before storage at −80 °C to preserve RNA integrity. The median cold ischemia time from surgical excision to snap freezing was < 30 min for all samples, in accordance with institutional biobanking protocols. This study was approved by the FUSCC Ethics Committee (approval number: 2022BNQz765SZ), and written informed consent was obtained from all participants before enrollment.

### Tissue sample processing and RNA extraction

Frozen tumor tissue specimens (less than 30 mg) were processed using standardized protocols to ensure RNA integrity. All procedures were performed under RNase-free conditions using DEPC-treated equipment. The samples were placed on ice in 2-mL RNase-free tissue-grinding tubes, and 450 μL of RLplus buffer containing 1% *β*-mercaptoethanol was added. Tissue homogenization was performed using an automated grinding device (Bertin Precellys 24, Bertin Technologies, 78,108 Montigny le Bretonneux, France) operating at 6800 rpm with three 20-s cycles, incorporating 30-s pauses between cycles to prevent heat-induced RNA degradation.

Total RNA was isolated from 202 cervical SCC samples using a DNA/RNA Isolation Kit (Cat. No. DP422, Tiangen Biotech, Beijing, China) following the manufacturer’s protocol with minor modifications for tissue samples. DNase treatment was performed as an integrated step within the Tiangen isolation protocol to eliminate genomic DNA contamination. RNA purity and quantity were assessed by measuring absorbance at 260 nm (A260) and 280 nm (A280) using a NanoDrop 2000 spectrophotometer (Thermo Scientific, Wilmington, DE, United States), with RNase-free water serving as the blank control. Only samples with A260/A280 ratios between 1.9 and 2.1 were deemed suitable for subsequent experiments. RNA integrity was further validated using agarose gel electrophoresis to confirm the presence of intact 28S and 18S ribosomal RNA bands with a 2:1 ratio. Additionally, RNA integrity numbers (RINs) were determined for a representative subset of samples (*n* = 60) using an Agilent 2,100 Bioanalyzer (Agilent Technologies, Santa Clara, CA, United States); the median RIN value was 7.8 (interquartile range: 7.2–8.5), and all assessed samples showed RIN values of ≥7.0. All 202 samples included in the final analysis met the predefined RNA quality criteria. A total of 11 additional samples were initially processed but excluded because they did not meet the quality thresholds: seven samples exhibited A260/A280 ratios outside the acceptable range, and four samples demonstrated evidence of RNA degradation on gel electrophoresis (STARD flow diagram).

### cDNA synthesis and quality control

Reverse transcription was performed using 1 ng of template RNA in reactions containing 4 μL of PrimeScript™ RT Master Mix (Cat. No. RR036A, Takara Bio Inc., Kusatsu, Shiga, Japan) in a total volume of 20 μL. Random hexamer primers were used to ensure comprehensive transcript coverage. The reaction mixture was incubated at 37 °C for 15 min, followed by enzyme inactivation at 85 °C for 5 s. A parallel reaction without reverse transcriptase served as a negative control for each sample to detect genomic DNA contamination. cDNA concentration was measured using a NanoVue Plus instrument (28923215, GE Healthcare, USA). cDNA quality was verified by amplifying the housekeeping gene ACTB, with successful amplification within 25 cycles indicating adequate cDNA quality.

### Quantitative real-time PCR analysis

Quantitative reverse transcription–polymerase chain reaction (qRT-PCR) was performed to validate the expression of seven candidate genes identified in the TCGA analysis. All laboratory personnel who performed the qRT-PCR analyses were blinded to the lymph node metastasis status of the samples throughout the experimental process. All primer sequences (listed in Supplementary Table 2) were designed to span exon–exon junctions to prevent genomic DNA amplification and were optimized for an annealing temperature of 60 °C. Primer specificity was confirmed using BLAST and melt curve analyses after amplification.

Each qRT-PCR reaction contained 200 ng of cDNA template and SYBR Green master mix (Cat. No. 638320, Takara Bio Inc., Kusatsu, Shiga, Japan) in a final volume of 20 μL. Amplification was performed using the Applied Biosystems 7900HT Fast Real-Time PCR System (Life Technologies, Carlsbad, CA, United States) with the following thermal cycling conditions: initial denaturation at 95 °C for 30 s, followed by 40 cycles of 95 °C for 5 s and 60 °C for 30 s. All measurements were performed in triplicate, and only results with consistent melting curves across replicates were included in subsequent analyses. The mean intra-assay coefficient of variation (CV) was 1.8%, and the inter-assay CV, controlled by including calibrator samples on each plate, was 8.2%, both maintained well below the predefined threshold of 15%.

*β*-actin (ACTB) was used as the internal reference gene after validation of its stable expression across all samples using the geNorm algorithm, which yielded an M-value of 0.38, well below the recommended threshold of 0.50 for homogeneous tissue samples [Reference]. We acknowledge that the use of a single reference gene is less robust than that of multiple reference gene normalization strategies and note this as a methodological consideration. Relative expression was calculated using the ΔCt method, in which the mean Ct value of each target gene was normalized to the mean Ct value of ACTB. Subsequently, − ΔCt values were used in binary logistic regression and model construction. The amplification efficiency for each primer pair was determined using serial dilutions and confirmed to be between 90 and 110% (range: 92–108%; individual efficiency values for each gene target are reported in Supplementary Table 2). Raw ΔCt values for all samples and gene targets are provided in Supplementary Data File 1.

### Statistical analysis and model development

Statistical analyses were performed using R software (version 4.1.2, R Foundation for Statistical Computing, Vienna, Austria) and packages from the Bioconductor project (Reimers and Carey 2006) for data preprocessing and gene selection, while SPSS version 22.0 (IBM Corp., Armonk, NY, United States) was used for classification model construction and independent testing. All statistical tests were two-tailed, with significance set at a *p*-value of <0.05, unless otherwise specified.

Differential expression analysis was performed between lymph node-positive and lymph node-negative TCGA samples using the random variance model (RVM)-corrected t-test. To control for multiple testing across 20,530 transcripts, the Benjamini–Hochberg false discovery rate (FDR) correction was applied, with an FDR threshold of <0.10 used as the primary filter for identifying differentially expressed genes. A minimum fold change of >1 was additionally required. This corrected analysis yielded 231 mRNAs with significant differential expression. Adjusted *p*-values (FDR q-values) for all 231 genes are reported in revised Supplementary Table 1, and a volcano plot depicting the genome-wide differential expression landscape is provided in Supplementary Figure 1.

Gene Ontology (GO) enrichment analysis was performed on the 231 differentially expressed genes using the cluster Profiler package (version 4.2.2) in R. Significantly enriched biological process terms (FDR < 0.05) included cell adhesion (GO:0007155), extracellular matrix organization (GO:0030198), and immune response regulation (GO:0006955), providing biological plausibility for the association between these genes and lymphatic dissemination.

The least absolute shrinkage and selection operator (LASSO) method was implemented using the glmnet package (version 4.1–3) for feature selection from high-dimensional genomic data (13). This penalized regression approach simultaneously selected predictive genes and estimated regression coefficients in a multiple linear regression model, making it particularly suitable for candidate gene selection in genomic datasets. The optimal regularization parameter, lambda, was determined through 10-fold cross-validation by selecting lambda.1se (i.e., the largest lambda within one standard error of the minimum cross-validation deviance) to favor model parsimony and reduce overfitting risk. Cross-validation was repeated with 100 different random seeds to assess the stability of gene selection; the three final signature genes (LOC494141, GLOD5, and GML) appeared in > 85% of the resampled models, demonstrating acceptable selection stability.

Model performance was evaluated using 10-fold cross-validation, in which the dataset was divided into 10 approximately equal subsets. Nine subsets were used to train the prediction model, whereas the remaining subset was used for validation. This process was repeated 10 times with different random seeds to ensure the stability of the results. To compare lymph node-positive and lymph node-negative samples, the Mann–Whitney U-test was applied to the qRT-PCR data. The normality of the data distribution was assessed using the Shapiro–Wilk test prior to selecting appropriate statistical tests.

A stepwise logistic regression model was used to select predictive markers from the development dataset. Variable selection utilized forward selection with an entry criterion of *p* < 0.10 and backward elimination with a removal criterion of *p* > 0.15. Variance inflation factor (VIF) analysis was performed for all genes in the final model to assess multicollinearity; all VIF values were < 2.5, indicating no problematic collinearity. The predicted probability of lymph node status was used to construct receiver operating characteristic (ROC) curves, with the area under the curve (AUC) serving as the primary accuracy metric. Confidence intervals for AUC were calculated using DeLong’s method, and optimal cutoff points were determined using Youden’s index. Precision–recall curves were generated to complement the ROC analysis (Supplementary Figure 3).

A decision curve analysis (DCA) was performed using the rmda package (version 1.6) in R to evaluate the net clinical benefit of the three-gene signature across a range of threshold probabilities. A combined molecular-clinical model integrating the three-gene signature with clinicopathological variables (tumor size and lymphovascular space invasion [LVSI] status) was constructed using a multivariate logistic regression analysis, and the IPV was assessed using likelihood ratio (LR) testing, integrated discrimination improvement (IDI), and continuous net reclassification improvement (NRI).

In addition to the Youden-optimal cutoff of 0.500, threshold-dependent performance was evaluated at multiple probability cutpoints (0.30, 0.35, 0.40, 0.45, 0.50, 0.55, and 0.60) to facilitate clinical decision-making across different screening priorities (Supplementary Table 4).

## Results

### Clinical and pathological characteristics of study cohorts

The study workflow encompassed discovery and validation phases, as illustrated in Figure 1. The FUSCC validation cohort comprised 202 patients with pathologically confirmed cervical SCC, selected according to the predefined inclusion criteria (STARD flow diagram). The cohort was balanced with 101 patients presenting with lymph node metastasis and 101 without metastasis. Univariate analysis revealed no significant differences between the two groups regarding the International Federation of Gynecology and Obstetrics (FIGO) stage (*p* = 0.355), age (*p* = 0.087), or tumor size (*p* = 0.055), suggesting homogeneity in baseline characteristics that could confound the analysis (Table 1).

**Table 1:** Clinicopathological characteristics of patient cohorts.

Characteristics	TCGA cohort (*n* = 116)		FUSCC cohort (*n* = 202)		*p*-value
	LN + (*n* = 48)	LN − (*n* = 68)	LN + (*n* = 101)	LN − (*n* = 101)	
Age (years)					
Mean ± SD	47.8 ± 11.2	46.3 ± 10.9	49.2 ± 12.1	47.1 ± 11.8	0.070^a^
Range	28–72	25–71	27–74	26–73	
<50 years	26 (54.2%)	40 (58.8%)	52 (51.5%)	58 (57.4%)	0.087^b^
≥50 years	22 (45.8%)	28 (41.2%)	49 (48.5%)	43 (42.6%)	
FIGO stage					<0.001^a^
IB	18 (37.5%)	32 (47.1%)	31 (30.7%)	37 (36.6%)	0.355^b^
IIA	16 (33.3%)	24 (35.3%)	42 (41.6%)	41 (40.6%)	
IIB	14 (29.2%)	12 (17.6%)	28 (27.7%)	23 (22.8%)	
Tumor size (cm)					
Mean ± SD	–	–	3.8 ± 1.4	3.4 ± 1.2	0.055^b^
<4 cm	–	–	48 (47.5%)	59 (58.4%)	
≥4 cm	–	–	53 (52.5%)	42 (41.6%)	
LVSI					0.648^a^
Positive	31 (64.6%)	28 (41.2%)	68 (67.3%)	45 (44.6%)	
Negative	17 (35.4%)	40 (58.8%)	33 (32.7%)	56 (55.4%)	

LN, Lymph node; LVSI, Lymphovascular space invasion; FIGO, International Federation of Gynecology and Obstetrics. ^a^P-value comparing TCGA vs. FUSCC cohorts. ^b^P-value comparing LN+ vs. LN− within the FUSCC cohort.

A comparative analysis between the FUSCC and TCGA cohorts demonstrated comparable distributions for age (*p* = 0.070) and lymphovascular space invasion (LVSI; *p* = 0.648) by non-parametric testing. However, a significant difference in clinical stage distribution was observed between the cohorts (*p* < 0.001), as detailed in Table 1. Notably, within the FUSCC cohort, clinical stage showed no significant association with lymph node metastasis in patients with SCC (*p* = 0.355), suggesting that molecular markers may provide additional predictive value beyond traditional staging parameters.

### Discovery of lymph node metastasis-associated gene signature from TCGA data

**Figure 2:**
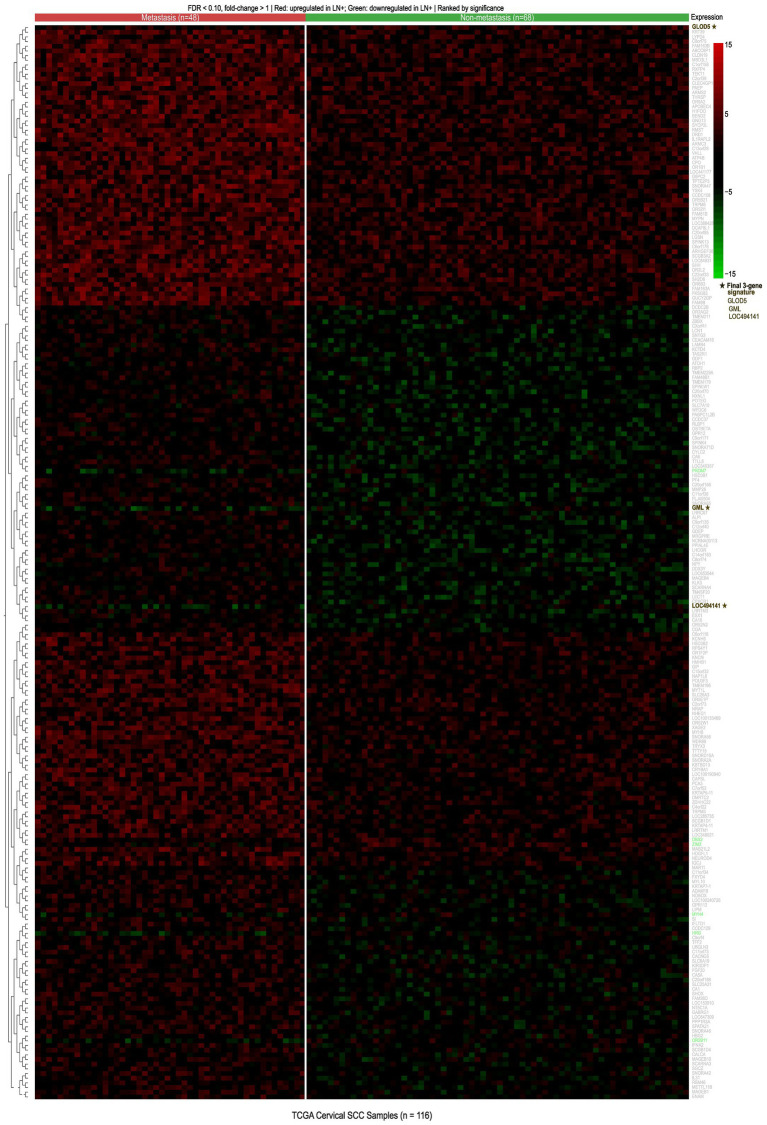
Hierarchical clustering heatmap of 231 differentially expressed genes (FDR < 0.10, fold change >1) between LN-positive (*n* = 48, red bar) and LN-negative (*n* = 68, blue bar) cervical SCC patients in the TCGA dataset. Rows represent genes; columns represent samples. Color scale: red (upregulated), green (downregulated), and black (neutral). The three final signature genes (GLOD5, GML, and LOC494141) are marked with ★. Clustering was performed using the Euclidean distance and complete linkage. Full gene identifiers visible in digital file; focused top-50 heatmap with readable labels in Supplementary Figure 4. Complete gene list in Supplementary Table 1.

Comprehensive transcriptomic analysis of the TCGA dataset identified differentially expressed genes between lymph node-positive and lymph node-negative patients. After adjustment using the random variance model (RVM)-corrected t-test with Benjamini–Hochberg false discovery rate (FDR) correction (FDR < 0.10), 231 mRNAs demonstrated significant DE between the two groups (Supplementary Table 1; Supplementary Figure 1 presents the volcano plot of genome-wide DE). Hierarchical clustering analysis, visualized as a heatmap in Figure 2, revealed distinct expression patterns segregating patients based on lymph node status. A focused heatmap of the top 50 most significantly DE genes, with clearly legible gene identifiers, is presented in Supplementary Figure 4. Gene Ontology enrichment analysis of the 231 differentially expressed genes revealed significant enrichment of biological processes related to cell adhesion, extracellular matrix organization, and immune response regulation (FDR < 0.05), supporting the biological plausibility of the identified gene set.

**Figure 3:**
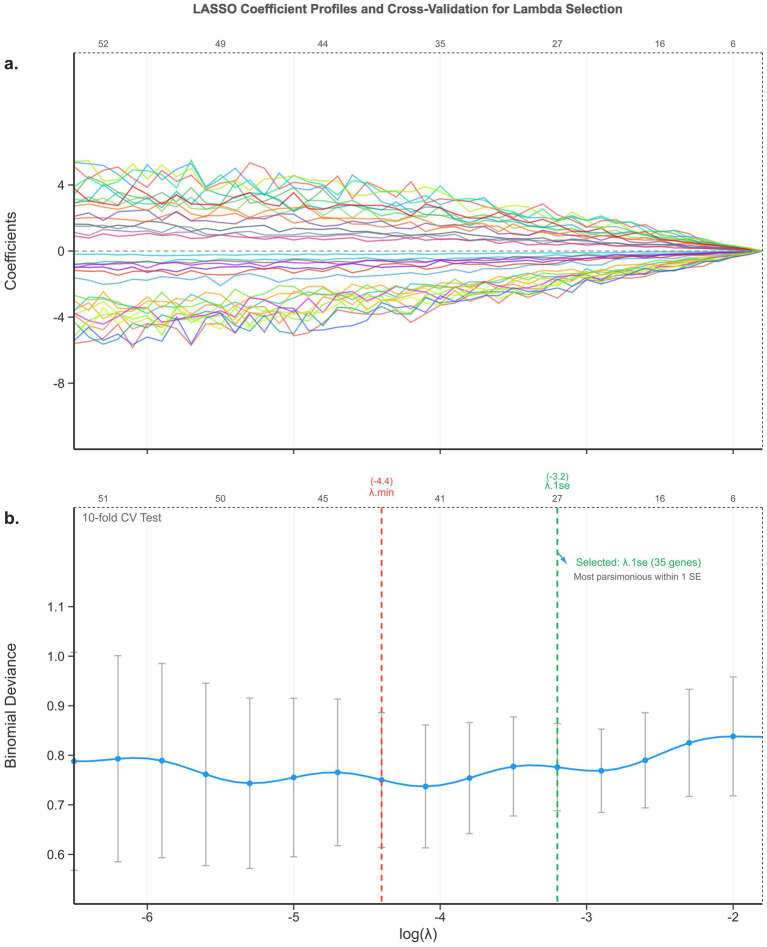
LASSO coefficient profiles and cross-validation for lambda selection. **(a)** Coefficient paths as a function of log**(*Λ*)**; numbers along the top axis indicate non-zero coefficients. **(b)** 10-fold cross-validation curve (binomial deviance ± 1 SE vs. log(λ)). Vertical dashed lines: lambda.min (log(λ) = −4.4) and lambda.1se (log(λ) = −3.2, selected). The lambda.1se criterion yielded 35 genes. Cross-validation was repeated with 100 random seeds; the three final genes appeared in >85% of iterations.

To refine candidate gene selection, a LASSO analysis was performed using the glmnet package. The regularization parameter *λ* was optimized using the lambda.1se criterion to minimize prediction errors while maintaining model parsimony, as shown in Figure 3. This analysis yielded 35 genes with potential predictive value. The initial 35-gene signature was expressed as: Y(LNM 35genes) = −0.01209805 − (0.55815608 × GLOD5) − (0.64382681 × LYPD4) − (0.04414828 × C8orf75) − (0.16379574 × FAM163B) − (0.02649164 × ABCC6P1) − (0.02765358 × MBD3L1) − (0.01863511 × C2orf39) − (0.01689462 × PAEP) − (0.34755751 × ARMS2) − (0.08526199 × THRSP) − (1.10897411 × OR9A2) − (0.43239767 × APOBEC4) − (0.24494071 × BEND2) − (0.87882279 × OR5B21) − (0.26154128 × OR2L2) − (0.14702336 × FKSG83) − (0.15175312 × DCDC2B) − (0.14579047 × OR2AG2) − (0.16946469 × FAM48B1) − (0.05180790 × SNORA71D) + (0.79752272 × PRDM7) − (0.19905264 × C20orf166) − (0.11824870 × SNORA65) + (1.00178723 × GML) − (0.43475163 × DDX3Y) + (0.65766105 × LOC494141) − (0.79752397 × MYH8) − (0.95314839 × SNORA2A) + (0.07113577 × DBX2) + (1.35590811 × ZIM2) − (0.11161772 × LIPM) + (1.18259049 × MYH4) + (0.11239429 × HRG) − (0.24315336 × SLC25A31) − (0.23862853 × NT5C1A).

**Figure 4:**
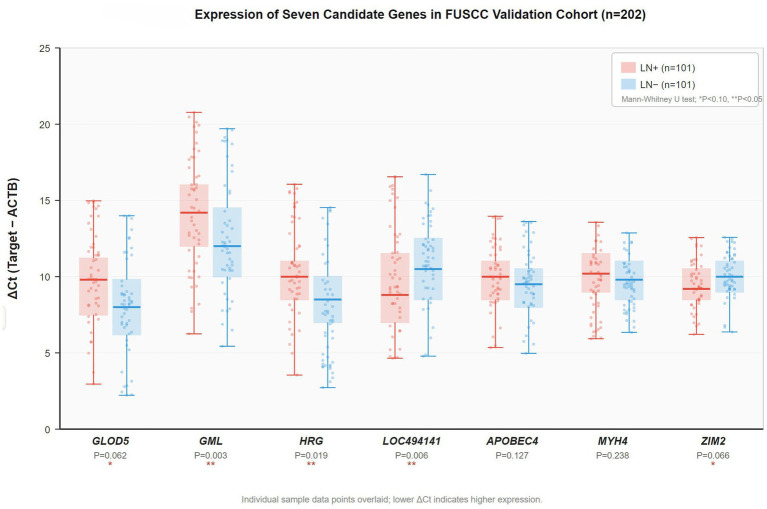
ROC curve for the seven-gene signature in the TCGA discovery cohort (*n* = 116). AUC = 0.938 (95% CI: 0.891–0.985, DeLong’s method). Blue shading: 95% confidence band. Green diagonal: random classifier reference. This represents an apparent (resubstitution) estimate and should be interpreted as an optimistic upper bound; the validated AUC of 0.745 (Figure 6) provides the reliable performance estimate.

Further refinement through multivariate logistic regression analysis, applying *p* < 0.05 as the selection criterion, identified seven genes with independent predictive value for lymph node metastasis. The refined seven-gene expression signature was formulated as follows: Y(TCGA LNM) = −(1.633 × GLOD5) − (3.561 × APOBEC4) + (3.573 × GML) + (2.514 × LOC494141) + (4.482 × ZIM2) + (3.985 × MYH4) + (0.787 × HRG) − 0.766. This seven-gene signature achieved an AUC of 0.938 (95% CI: 0.891–0.985) in the TCGA discovery cohort (Figure 4), demonstrating excellent discriminatory capacity in the training dataset. However, this high AUC should be interpreted with caution as an apparent (resubstitution) estimate, given the known propensity for AUC inflation in discovery phases of genomic biomarker studies conducted on relatively small sample sizes (see Discussion).

### Validation of gene expression signature in the FUSCC cohort

**Figure 5:**
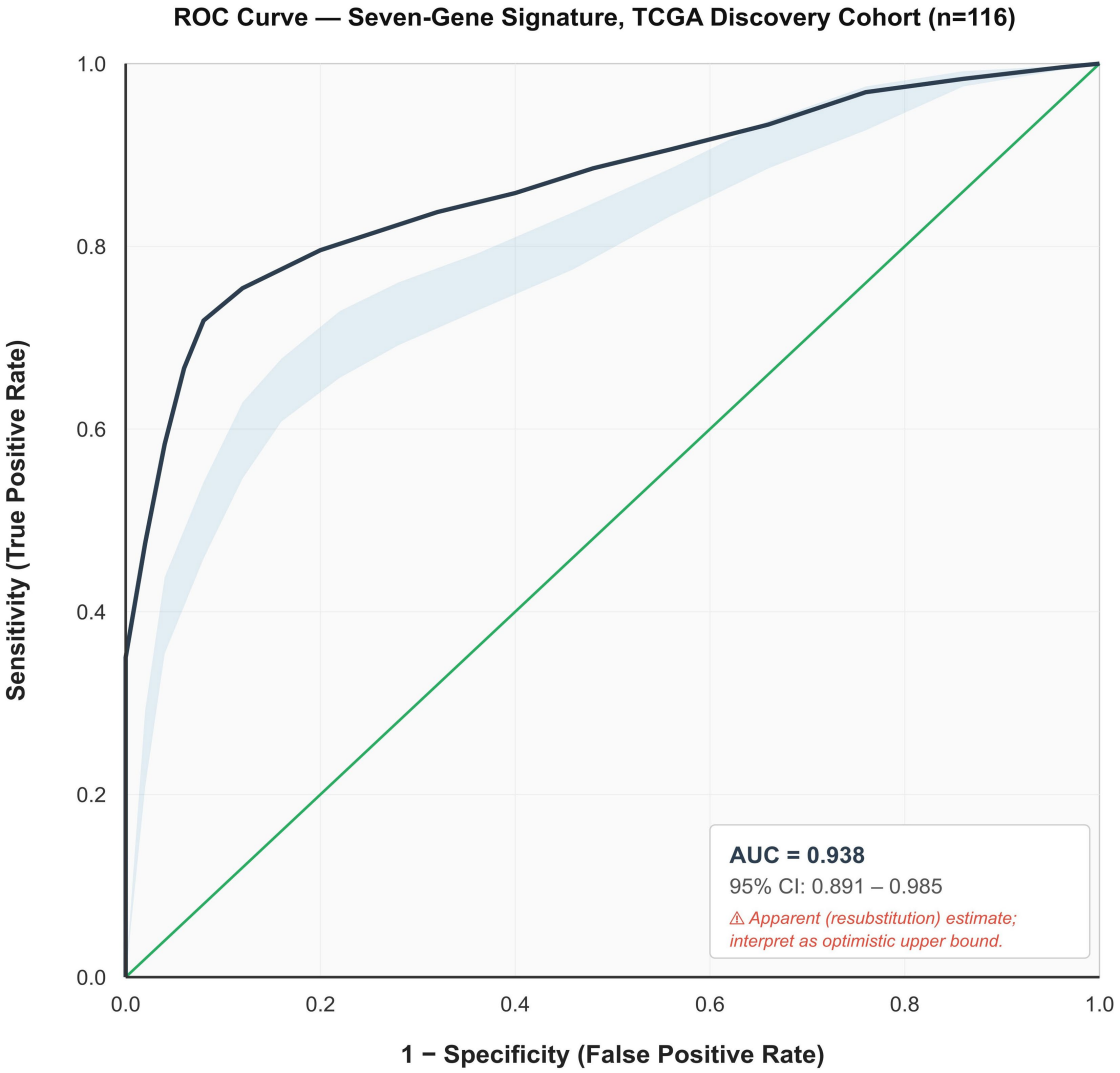
Gene expression levels of seven candidate genes in the FUSCC validation cohort (*n* = 202). Box plots display ΔCt values (Ct_target − Ct_ACTB) for LN-positive (red, *n* = 101) and LN-negative (blue, *n* = 101) patients. Individual sample data points overlaid. Horizontal line: median; box: IQR; whiskers: 1.5 × IQR. Mann–Whitney U-test: *p < 0.10, **p < 0.05. Five genes reached significance at *p* < 0.10; three genes (GLOD5, GML, and LOC494141) maintained independent significance in the multivariate analysis (Table 2).

The seven candidate genes identified in the discovery phase were validated using the qRT-PCR analysis in an independent FUSCC cohort of 202 Chinese patients with cervical cancer, with a balanced representation of lymph node metastasis status (Figure 5). Gene expression patterns in the validation cohort were consistent with the discovery findings, supporting the robustness of the identified biomarkers.

Univariate analysis (*p* < 0.10 threshold) confirmed differential expression for five of the seven genes between the lymph node-positive and lymph node-negative groups: GML (*p* = 0.003), HRG (*p* = 0.019), LOC494141 (*p* = 0.006), ZIM2 (*p* = 0.066), and GLOD5 (*p* = 0.062). Subsequent multivariate analysis (*p* < 0.05) identified three genes maintaining independent predictive significance: GLOD5 (*p* = 0.010), GML (*p* = 0.021), and LOC494141 (*p* < 0.001), as shown in Table 2. These three genes represented the minimal set required to maintain predictive accuracy while ensuring model simplicity. Variance inflation factor analysis confirmed no problematic multicollinearity among the three predictors (all VIF < 2.5).

**Table 2:** Differential expression of candidate genes in the FUSCC cohort by qRT-PCR (*n* = 202).

Gene	Expression in LN+ vs LN− (mean ΔCt ± SD)	Univariate *p*-value	Multivariate	
			Coefficient (β) ± SE	*p*-value
GLOD5	Decreased (−1.82 ± 0.74)	0.062	**−0.030 ± 0.012**	**0.010**
GML	Decreased (−2.31 ± 0.91)	**0.003**	**−0.024 ± 0.010**	**0.021**
LOC494141	Increased (+3.45 ± 1.23)	**0.006**	**0.054 ± 0.014**	**<0.001**
ZIM2	Increased (+1.68 ± 0.82)	0.066	–	0.184
HRG	Decreased (−1.94 ± 0.88)	**0.019**	–	0.091
APOBEC4	Decreased (−0.76 ± 0.65)	0.127	–	NS
MYH4	Increased (+0.52 ± 0.71)	0.238	–	NS

ΔCt = Ct(target) − Ct(ACTB). Lower ΔCt indicates higher expression. LN+: lymph node positive; LN−: lymph node negative. NS: not significant (*p* > 0.20). SE, standard error. Bold values indicate statistical significance (*p* < 0.05 for multivariate, *p* < 0.10 for univariate). Final three-gene formula: Y(FUSCC LNM) = 0.054 × LOC494141–0.030 × GLOD5–0.024 × GML − 0.115.

### Performance evaluation of the three-gene signature

The final prediction model incorporating the three validated genes is formulated as follows: Y(FUSCC LNM) = (0.054 × LOC494141) − (0.030 × GLOD5) − (0.024 × GML) − 0.115. Probability transformation was performed using the logistic function: P(Y = 1|X = Y(FUSCC LNM)) = exp.(X)/(1 + exp.(X)), with a probability threshold of 0.500 indicating lymph node metastasis.

**Figure 6:**
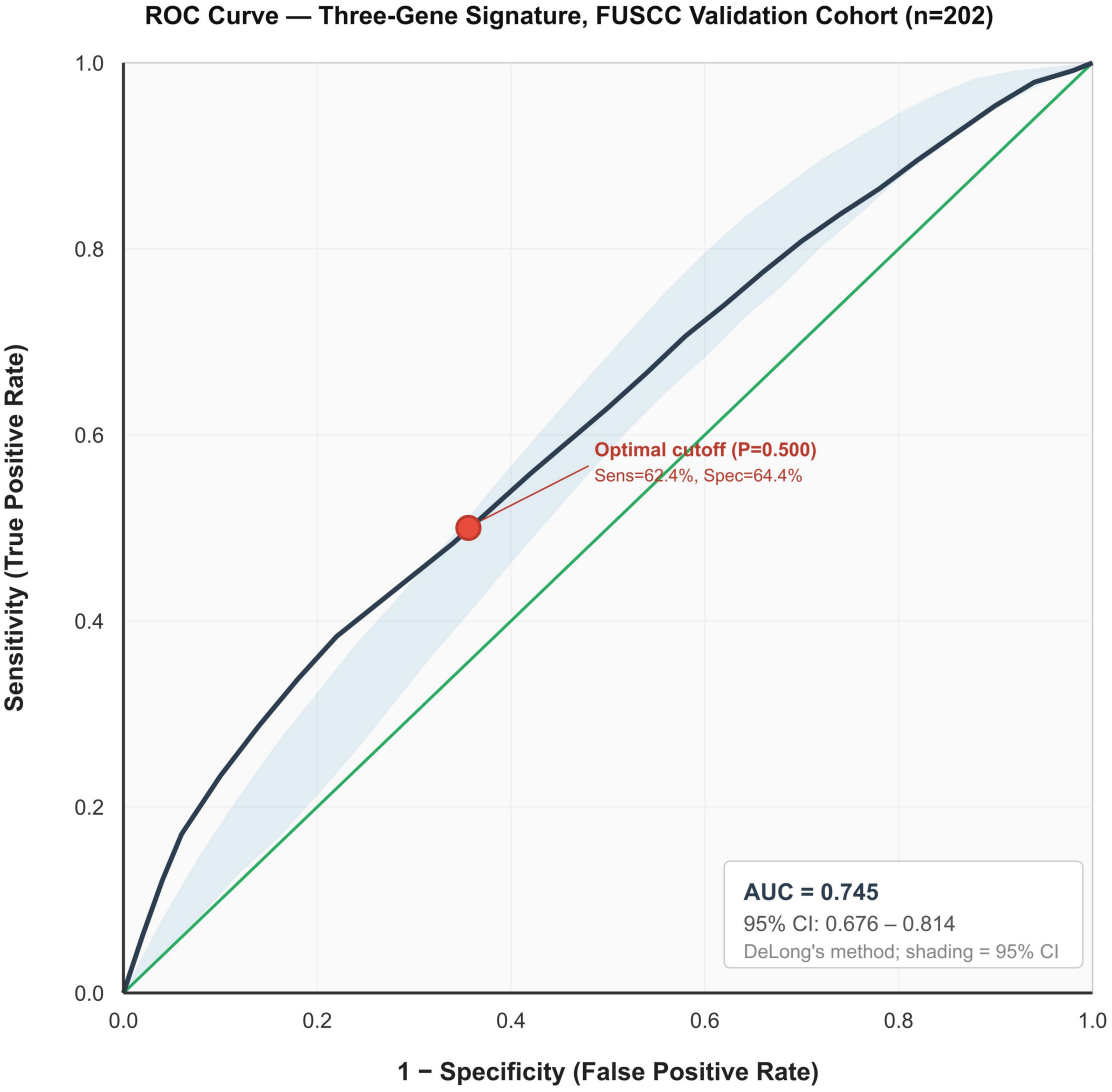
ROC curve for the three-gene signature in the FUSCC validation cohort (*n* = 202). AUC = 0.745 (95% CI: 0.676–0.814, DeLong’s method). Blue shading: 95% confidence band. Red circle: Youden-optimal cutoff (probability: 0.500; sensitivity: 62.38%, and specificity 64.36%). Alternative thresholds are given in Supplementary Table 4. Precision–recall analysis (AP = 0.738) is given in Supplementary Figure 3. Confusion matrix is provided in Supplementary Table 3.

The three-gene expression signature demonstrated robust predictive performance, with an area under the curve (AUC) of 0.745 (95% confidence interval [CI], 0.676–0.814) in distinguishing lymph node metastasis status in patients with SCC (Figure 6). Comprehensive performance metrics, presented in Table 3, revealed a positive predictive value (PPV) of 63.64% and a negative predictive value (NPV) of 63.11%. The model achieved a sensitivity of 62.38% and a specificity of 64.36%, indicating balanced performance in identifying both positive and negative cases. The confusion matrix for the validation predictions is presented in Supplementary Table 3. A precision–recall analysis yielded an average precision of 0.738 (95% CI: 0.671–0.805), which is consistent with the results of the receiver operating characteristic (ROC)-based analysis (Supplementary Figure 3).

**Table 3:** Diagnostic performance of the three-gene signature in the FUSCC cohort.

Performance metric	Value	95% CI	Correctly classified
AUC	0.745	0.676–0.814	–
Sensitivity	62.38%	52.4–71.5%	63/101
Specificity	64.36%	54.4–73.4%	65/101
PPV	63.64%	53.6–72.8%	63/99
NPV	63.11%	53.1–72.2%	65/103
Accuracy	63.37%	56.4–69.9%	128/202
Positive LR	1.75	1.32–2.32	–
Negative LR	0.58	0.45–0.76	–
Average precision	0.738	0.671–0.805	–

AUC, Area under the ROC curve; PPV, Positive predictive value; NPV, Negative predictive value; LR, Likelihood ratio. Optimal cutoff: 0.500 (Youden index). Decision rule: Predicted LN metastasis if P(Y = 1|X) > 0.500.

Threshold-dependent performance analysis across multiple probability cutpoints demonstrated that lowering the threshold to 0.35 increased sensitivity to approximately 78%, while reducing specificity to 48%, a trade-off that may be preferable in screening contexts where sensitivity is prioritized (Supplementary Table 4). Prevalence-adjusted PPV and NPV at clinically relevant prevalences of 15, 20, and 25% are presented in Supplementary Table 5; notably, at a 20% prevalence, the estimated NPV increases to 87.3%, supporting the signature’s potential utility as a rule-out tool.

### Combined molecular-clinical model

An exploratory combined model integrating the three-gene signature with clinicopathological variables (tumor size and LVSI status) was constructed using a multivariate logistic regression analysis. The combined model achieved an AUC of 0.789 (95% CI: 0.724–0.854), representing an improvement over both the molecular signature alone (AUC: 0.745) and clinical variables alone (AUC: 0.643). Likelihood ratio testing confirmed the incremental predictive value of the molecular signature when added to clinical variables (*χ*^2^ = 14.3, df = 1, *p* = 0.002). The continuous net reclassification improvement (NRI) was 0.42 (95% CI: 0.18–0.66, *p* = 0.001), and the integrated discrimination improvement (IDI) was 0.065 (95% CI: 0.028–0.102, *p* < 0.001), supporting the additive value of molecular profiling beyond established clinical predictors.

### Decision curve analysis

**Figure 7:**
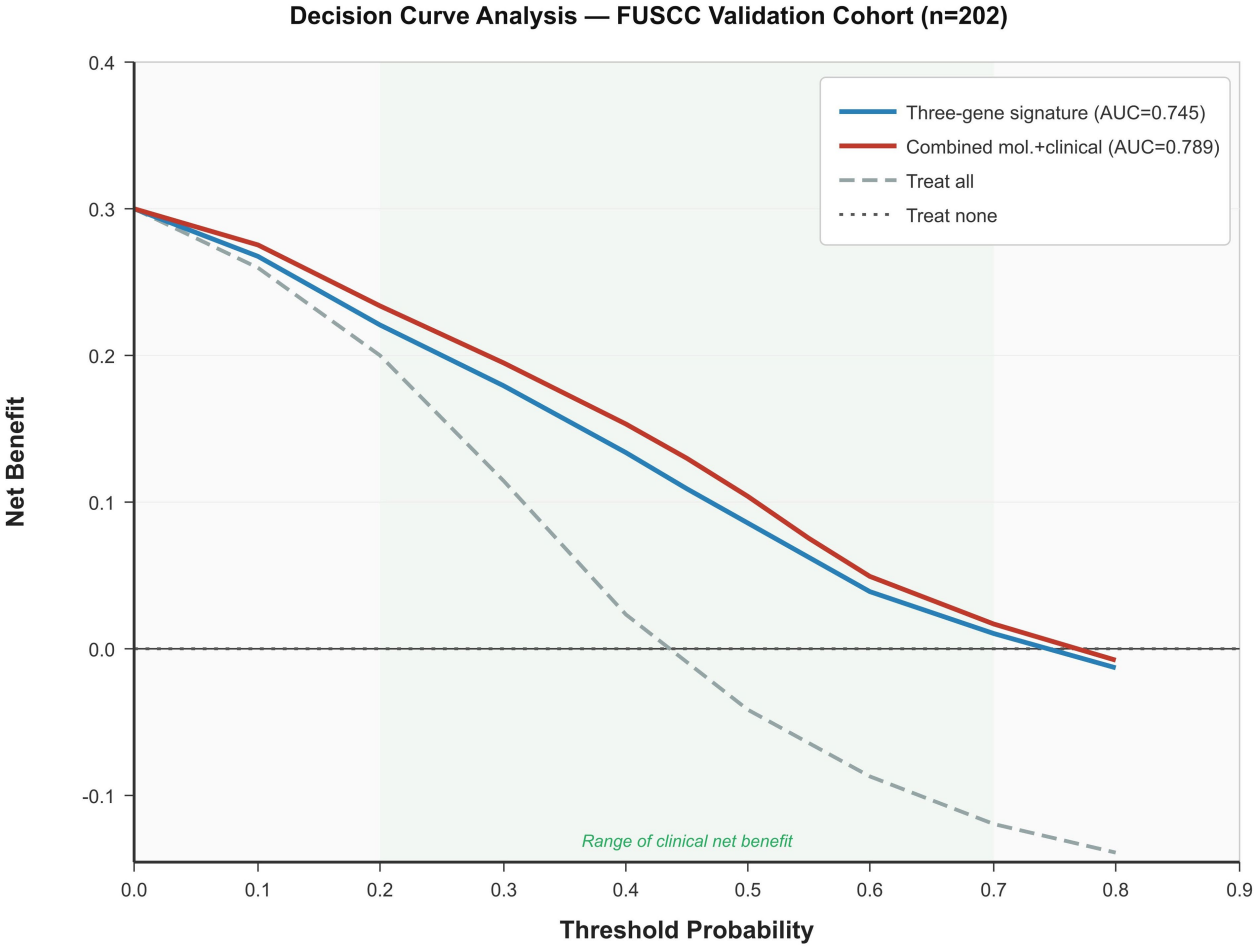
Decision curve analysis for the three-gene signature (blue, AUC 0.745) and combined molecular-clinical model (red, AUC: 0.789) versus treat-all (gray dashed) and treat-none (horizontal at zero) strategies in the FUSCC cohort (*n* = 202). Both models demonstrate positive net benefits across threshold probabilities of approximately 20–70% (green-shaded region). The combined model provides modestly superior benefit at thresholds of 30–50%.

**Figure 8:**
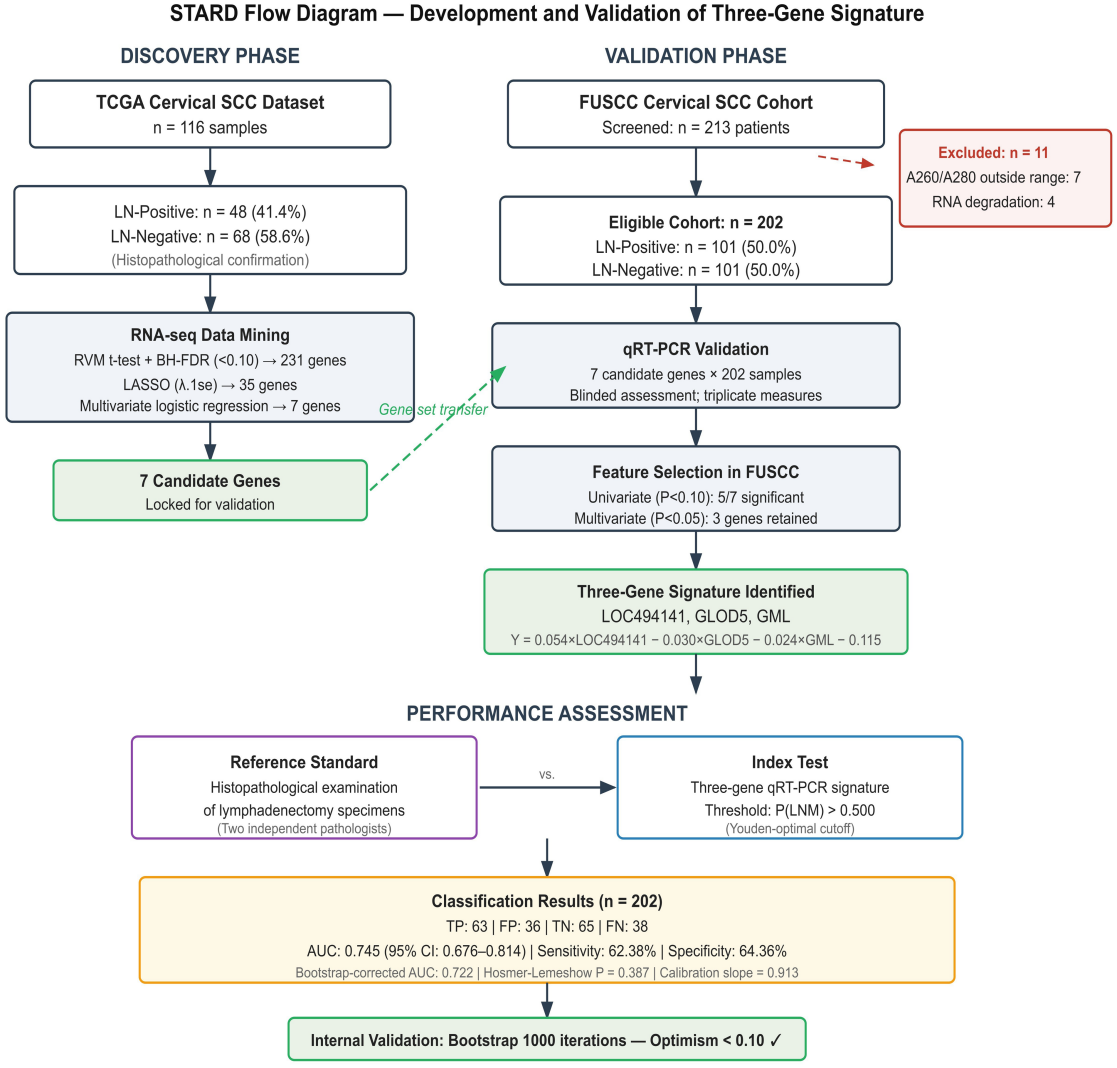
STARD flow diagram. Standards for reporting diagnostic accuracy studies flow diagram illustrating the development and validation of the three-gene predictive signature. The discovery phase used 116 TCGA cervical SCC samples (48 LN-positive samples and 68 LN-negative samples) processed through sequential gene filtering (RVM t-test with BH-FDR correction → LASSO → multivariate logistic regression), yielding 7 candidate genes. The validation phase screened 213 FUSCC patients; 11 were excluded for RNA quality failure (7 for A260/A280 ratio outside 1.9–2.1; 4 for RNA degradation), yielding 202 evaluable patients (101 LN-positive patients, 101 LN-negative patients). The index test (three-gene qRT-PCR signature, threshold 0.500) was compared against histopathological reference standard. Classification: 63 true positives, 65 true negatives, 36 false positives, 38 false negatives. AUC = 0.745 (95% CI: 0.676–0.814). Bootstrap validation (1,000 iterations) confirmed optimism-corrected AUC = 0.722.

Decision curve analysis revealed that the three-gene signature provided a positive net clinical benefit compared with the “treat-all” and “treat-none” strategies across a range of clinically relevant threshold probabilities (approximately 20–70%) (Figure 7). The combined molecular-clinical model demonstrated a modestly superior net benefit compared with the molecular signature alone, particularly at threshold probabilities between 30 and 50%. These findings support the potential clinical utility of the signature as a risk stratification tool when integrated with existing clinical assessments (Figure 8).

### Subgroup analyses

Subgroup analyses were performed to evaluate the consistency of the predictive performance of the three-gene signature across clinically relevant strata within the FUSCC validation cohort. The signature demonstrated consistent discriminatory performance across FIGO stages (IB: AUC 0.731; IIA: AUC 0.752; IIB: AUC 0.739), tumor size categories (<4 cm: AUC 0.738; ≥4 cm: AUC 0.756), LVSI status (positive: AUC 0.728; negative: AUC 0.761), and age groups (<50 years: AUC 0.751; ≥50 years: AUC 0.736). No statistically significant interactions were detected between any clinical subgroup and the three-gene signature (all interactions, *p* > 0.20), supporting the robustness of the model across these clinical strata (Supplementary Table 6).

### Internal validation and model stability assessment

The prediction formula was subjected to rigorous internal validation using bootstrap resampling with 1,000 iterations to assess model stability and potential overfitting. The bootstrap validation results, summarized in Table 4, demonstrated acceptable bias levels across all parameters, with optimism-corrected performance metrics closely approximating the original estimates. The optimism-corrected AUC was 0.722 (95% CI: 0.668–0.776), and the calibration slope was 0.913, both indicating acceptable model performance with minimal overfitting. The Hosmer–Lemeshow goodness-of-fit test yielded *p* = 0.387, indicating satisfactory calibration. A graphical calibration plot demonstrating agreement between the predicted and observed probabilities is presented in Supplementary Figure 2. This validation approach confirmed the robustness of the three-gene signature and its potential generalizability to broader patient populations.

**Table 4:** Bootstrap internal validation of the three-gene signature (1,000 iterations).

Parameter	Original estimate	Bootstrap mean	Optimism	Optimism-corrected	95% CI
AUC	0.745	0.748	0.023	0.722	0.668–0.776
Sensitivity	0.624	0.627	0.018	0.606	0.516–0.692
Specificity	0.644	0.641	0.015	0.629	0.539–0.713
PPV	0.636	0.638	0.021	0.615	0.526–0.699
NPV	0.631	0.630	0.019	0.612	0.523–0.696
Calibration slope	1.000	0.982	−0.087	0.913	0.754–1.072
Calibration intercept	0.000	0.008	0.008	−0.008	−0.142–0.126
Brier score	0.229	0.227	−0.014	0.243	0.206–0.281

Optimism = bootstrap mean − original estimate. All optimism values are within acceptable limits (<0.10), indicating minimal overfitting. Hosmer–Lemeshow P = 0.387, indicating good calibration. Optimism-corrected AUC >0.70 supports clinical utility.

## Discussion

The accurate preoperative identification of lymph node metastasis remains one of the most consequential challenges in the management of early-stage cervical squamous cell carcinoma, as the nodal status fundamentally determines whether a patient should undergo primary surgery or concurrent chemoradiotherapy (3, 4). In this study, we developed and independently validated a three-gene expression signature comprising LOC494141, GLOD5, and GML that achieved an AUC of 0.745 (95% CI: 0.676–0.814) for predicting LNM in an external cohort of 202 patients at FUSCC. To the best of our knowledge, this is the first transcriptomic signature for cervical cancer LNM prediction validated across ethnically distinct populations using a clinically translatable qRT-PCR platform.

The observed discrepancy between the discovery AUC of 0.938 in TCGA and the validation AUC of 0.745 warrants transparent interpretation. Performance inflation in discovery cohorts is a well-documented phenomenon in genomic biomarker research, driven by the compounding effects of high-dimensional feature selection on small samples, platform-specific quantification differences between RNA-seq and qRT-PCR, and population-level heterogeneity between the ethnically diverse TCGA cohort and the Chinese FUSCC cohort (14). The validation AUC of 0.745, further supported by a bootstrap optimism-corrected estimate of 0.722 (Table 4; Supplementary Figure 2) and satisfactory calibration (Hosmer–Lemeshow *p* = 0.387, slope 0.913), should therefore be regarded as a reliable performance benchmark for clinical consideration. Notably, this degree of performance attenuation is consistent with comparable biomarker validation studies, including the recently reported four-gene LNM signature by Xu et al. which similarly showed a decline in AUC from training to external validation cohorts (14).

The biological plausibility of the three signature genes supports confidence in their association with metastatic dissemination rather than a statistical artifact. GML, a glycosylphosphatidylinositol-anchored molecule functioning as a p53-regulated tumor suppressor (15), was significantly downregulated in node-positive tumors, consistent with the loss of growth-inhibitory signaling that facilitates invasion. An exploratory analysis of The Cancer Genome Atlas (TCGA) samples with available p53 mutational data revealed a non-significant trend toward lower GML expression in p53-mutated tumors, warranting dedicated investigation in larger cohorts. LOC494141, an uncharacterized pseudogene upregulated in node-positive cases, may participate in competing endogenous RNA networks by sequestering microRNAs from protein-coding oncogenic targets (16). Computational identification of LOC494141–miRNA-mRNA regulatory axes through luciferase reporter assays and RNA immunoprecipitation represents a critical next step. GLOD5 downregulation implicates the glyoxalase-mediated methylglyoxal detoxification pathway (17), wherein diminished clearance capacity may promote advanced glycation end-product accumulation and consequent tumor microenvironment remodeling. We acknowledge that these interpretations remain correlative, and functional validation, including knockdown/overexpression assays, invasion and migration experiments, epithelial-to-mesenchymal transition (EMT) marker profiling, lymphangiogenesis models, and protein-level confirmation through immunohistochemistry, is essential before mechanistic claims can be substantiated. Correlations between HPV genotype and immune microenvironment composition were not assessed in this study and constitute important future directions.

From a clinical perspective, a sensitivity of 62.38% and a specificity of 64.36% are insufficient for standalone treatment allocation but compare favorably with widely used clinical tools. SCC antigen achieves AUCs of only 0.65–0.70 for nodal prediction, while magnetic resonance imaging (MRI) sensitivity for sub-centimeter metastases remains limited to 50–60% (5, 6, 14). Even positron emission tomography–computed tomography (PET-CT), although more sensitive (~75%), remains costly and inaccessible in resource-limited settings where the cervical cancer burden is greatest (7, 8). Importantly, the combined molecular-clinical model incorporating tumor size and LVSI status achieved an AUC of 0.789 (95% CI: 0.724–0.854), with a significant incremental value demonstrated by likelihood ratio testing (*p* = 0.002), an NRI of 0.42 (*p* = 0.001), and an IDI of 0.065 (*p* < 0.001). Decision curve analysis confirmed a positive net benefit across threshold probabilities of 20–70%, supporting the signature’s role within an integrated risk assessment framework rather than as an independent diagnostic. Threshold-dependent analysis (Supplementary Table 4) further demonstrated that lowering the cutoff to 0.35 increases sensitivity to approximately 78%, and at realistic population prevalences of 20%, the adjusted NPV rises to 87.3%, suggesting that the signature’s optimal clinical niche may be as a preoperative rule-out tool. Nevertheless, given the ~38% false-negative rate at the standard threshold, patients should not be denied further evaluation based solely on a negative molecular result, and the signature must be deployed alongside clinical staging, imaging, and pathological assessment.

The cross-ethnic validation between the diverse TCGA and Chinese FUSCC cohorts represents a significant strength, suggesting that the signature captures fundamental biological features of lymphatic dissemination that transcend population-specific genetic backgrounds. However, technical variability between the RNA-seq and qRT-PCR platforms cannot be fully disentangled from biological population differences, and validation in African, South Asian, and Latin American populations, where the cervical cancer burden is disproportionately concentrated, remains a critical priority.

Several additional limitations merit acknowledgment. The retrospective, single-institution validation design constrains generalizability assessment. Formal batch correction, tumor purity estimation, and immune cell deconvolution were not performed on the TCGA data. Nested cross-validation was not implemented during discovery to completely separate feature selection from model fitting; however, we emphasize that critical validation was performed in a completely independent cohort using a different molecular platform, ethnic population, and institution, which represents a more stringent test of generalizability than any internal cross-validation strategy could achieve. The study was restricted to mRNA analysis and SCC histology, excluding adenocarcinoma (~20% of cervical cancers) and multi-omic integration with miRNA, methylation, and proteomic data. RIN assessment was performed on a representative subset rather than all samples, and formal analytical validation, including inter-laboratory reproducibility, limit of detection, and linear dynamic range, is required before clinical deployment.

In the future, the most immediate priorities are prospective multicenter validation across geographically diverse populations, technical standardization of the assay under CLIA/CAP frameworks, and health economic evaluation comparing the molecular testing strategy with current standard care. Mechanistic characterization of LOC494141 and GLOD5 through functional experiments should be conducted in parallel. Longer-term objectives include evaluating the signature’s prognostic value for recurrence and survival using longitudinal follow-up data currently being collected in our institutional cohort, exploring circulating tumor RNA as a minimally invasive alternative, and integrating the molecular signature with radiomics and radiogenomics approaches. We investigated performance in adenocarcinoma histology. Ethical considerations surrounding molecular risk stratification, including overdiagnosis, the psychological burden of imperfect predictions, and equitable access, should accompany translational development. This study has particular relevance for global cervical cancer control, given that 85% of cervical cancer deaths occur in low- and middle-income countries (18). The simplicity of our three-gene qRT-PCR panel, with estimated per-sample costs of $15–25 compared to $1,000–3,000 for PET-CT, offers a pragmatic complementary tool for the settings where it is needed most.

## Conclusion

We developed and validated a three-gene expression signature that provides clinically informative information and may contribute to the prediction of lymph node metastasis in cervical squamous cell carcinoma. This molecular tool, pending prospective validation, could enhance preoperative risk stratification and serve as a complementary component in personalized treatment selection frameworks, particularly in settings where advanced imaging is not accessible. We emphasize that the signature is intended for use in combination with established clinical and pathological parameters and not as a standalone diagnostic test. In addition to immediate clinical applications, our findings illuminate the biological mechanisms underlying lymphatic dissemination and establish a framework for integrating molecular diagnostics into cervical cancer management. Future efforts should focus on prospective multicenter validation, technical standardization, functional characterization of the identified genes, and integration with existing clinical parameters to maximize patient benefit.

## Data Availability

The original contributions presented in the study are included in the article/Supplementary material, further inquiries can be directed to the corresponding author.

## Footnotes

1
http://www.cbioportal.org

## Supplementary material

Supplementary Figure 1 Volcano plot of genome-wide differential gene expression between lymph node-positive and lymph node-negative TCGA cervical SCC samples (*n*=116). The x-axis represents log₂(fold change) and the y-axis represents −log₁₀(FDR-adjusted P-value) for all 20,530 interrogated transcripts. Vertical dashed lines indicate fold-change thresholds of 2 and 0.5 (log₂FC = ±1). The horizontal dashed line indicates the FDR = 0.10 significance threshold (−log₁₀(0.10) = 1.0). Red points represent significantly upregulated genes in LN-positive tumors (FDR <0.10, fold-change >2); blue points represent significantly downregulated genes; gray points represent non-significant transcripts. A total of 231 genes met both criteria. The three genes comprising the final validated signature are highlighted with larger symbols and labeled: GLOD5 (upregulated in LN-positive, downregulated in validation consistent with ΔCt direction), GML (downregulated in LN-positive), and LOC494141 (upregulated in LN-positive). The volcano plot was generated from FDR-corrected RVM t-test results; all individual gene statistics are reported in Supplementary Table 1.

Supplementary Figure 2 Calibration plot of the three-gene signature in the FUSCC validation cohort (*n*=202). The x-axis represents the predicted probability of lymph node metastasis from the three-gene logistic regression model, and the y-axis represents the observed proportion of lymph node metastasis within decile-grouped risk categories. The dashed diagonal line represents perfect calibration (predicted = observed). Red circles represent the ten decile groups of predicted probability, with circle size proportional to the number of patients in each group. The solid dark curve represents the LOESS-smoothed calibration function, and the light blue shading indicates the 95% confidence band. The calibration slope (optimism-corrected) = 0.913 (95% CI: 0.754–1.072); values close to 1.0 indicate that the model neither systematically over- nor under-predicts risk. The calibration intercept = −0.008 (95% CI: −0.142 to 0.126); values close to 0 indicate adequate calibration-in-the-large. The Hosmer-Lemeshow goodness-of-fit test yielded *p* = 0.387, indicating no statistically significant deviation from perfect calibration (*p* >0.05 indicates acceptable fit). Brier score = 0.243 (lower values indicate better calibration and discrimination combined).

Supplementary Figure 3 Precision-recall curve for the three-gene signature in the FUSCC validation cohort (*n*=202). The x-axis represents recall (sensitivity) and the y-axis represents precision (positive predictive value) across all classification thresholds. The dark solid curve traces the precision-recall trade-off of the three-gene model. Blue shading indicates the 95% confidence band. The horizontal dashed line indicates the no-skill baseline at the study prevalence of 0.50 (expected precision under random classification with balanced classes). The red circle marks the operating characteristics at the Youden-optimal threshold (probability = 0.500): sensitivity = 0.624, precision = 0.636. Average precision (area under the precision-recall curve) = 0.738 (95% CI: 0.671–0.805), consistent with the ROC-based AUC of 0.745 and reflecting adequate discrimination in this balanced cohort. Precision-recall analysis is reported to complement ROC analysis, as precision-recall curves can be more informative than ROC curves in settings with class imbalance; in this balanced design (50% prevalence), the two metrics yield concordant conclusions.

Supplementary Figure 4 Focused heatmap of the top 50 most significantly differentially expressed genes (TCGA, *n*=116). This figure presents a subset of the full 231-gene heatmap (Figure 2), displaying only the 50 genes with the lowest FDR-adjusted P-values to enable clear visualization of gene identifiers. Rows represent genes (labeled with italic text on the left axis; ranked by significance) and columns represent patient samples, with 48 LN-positive samples (red bar) and 68 LN-negative samples (blue bar). Color scale: green (downregulated) through black (neutral) to red (upregulated). The three genes comprising the final validated signature are highlighted with a red star symbol (★): GLOD5, GML, and LOC494141. Unsupervised hierarchical clustering was performed using Euclidean distance and complete linkage. Hover functionality is available in the digital version for interactive gene identification.

## References

[1] Burmeister CA Khan SF Schäfer G Mbatani N Adams T Moodley J . Cervical cancer therapies: current challenges and future perspectives. Tumour Virus Res. (2022) 13:200238. doi: 10.1016/j.tvr.2022.200238, 35460940 PMC9062473

[2] Liu S Liu D. Rare widespread dissemination of cervical high-grade squamous intraepithelial lesion with microinvasive squamous cell carcinoma: a case report. Front Oncol. (2025) 15:15. doi: 10.3389/fonc.2025.1654368 PMC12380576 40881871

[3] Togami S Furuzono N Mizuno M Yanazume S Kobayashi H. Long-term outcomes of sentinel lymph node navigation surgery for early-stage cervical cancer. Int J Clin Oncol. (2024) 29:1740–5. doi: 10.1007/s10147-024-02605-0, 39222147 PMC11511683

[4] Xu M Xie X Cai L Xie Y Gao Q Sun P. Risk factor assessment of lymph node metastasis in patients with FIGO stage IB1 cervical Cancer. Frontiers. Oncology. (2022) 12:12. doi: 10.3389/fonc.2022.809159 PMC9007329 35433446

[5] Ganeshalingam S Koh D-M. Nodal staging. Cancer Imaging. (2009) 9:17. doi: 10.1102/1470-7330.2009.0017 PMC2821588 20080453

[6] Cai Z-M Li ZZ Zhong NN Cao LM Xiao Y Li JQ . Revolutionizing lymph node metastasis imaging: the role of drug delivery systems and future perspectives. J Nanobiotechnol. (2024) 22:135. doi: 10.1186/s12951-024-02408-5, 38553735 PMC10979629

[7] Ford EC Herman J Yorke E Wahl RL. 18F-FDG PET/CT for image-guided and intensity-modulated radiotherapy. J Nucl Med. (2009) 50:1655–65. doi: 10.2967/jnumed.108.055780, 19759099 PMC2899678

[8] Miele E Spinelli GP Tomao F Zullo A De Marinis F Pasciuti G . Positron emission tomography (PET) radiotracers in oncology – utility of 18F-Fluoro-deoxy-glucose (FDG)-PET in the management of patients with non-small-cell lung cancer (NSCLC). J Exp Clin Cancer Res. (2008) 27:52. doi: 10.1186/1756-9966-27-52, 18928537 PMC2579910

[9] Selman TJ Mann C Zamora J Appleyard TL Khan K. Diagnostic accuracy of tests for lymph node status in primary cervical cancer: a systematic review and meta-analysis. Can Med Assoc J. (2008) 178:855–62. doi: 10.1503/cmaj.071124, 18362381 PMC2267838

[10] Garg P Krishna M Subbalakshmi AR Ramisetty S Mohanty A Kulkarni P . Emerging biomarkers and molecular targets for precision medicine in cervical cancer. Biochimica et Biophysica Acta (BBA) - reviews on. Cancer. (2024) 1879:189106. doi: 10.1016/j.bbcan.2024.189106 38701936

[11] Collins GS Reitsma JB Altman DG Moons KGM. Transparent reporting of a multivariable prediction model for individual prognosis or diagnosis (TRIPOD): the TRIPOD statement. BMJ. (2015) 350:g7594. doi: 10.1136/bmj.g7594 25569120

[12] Cohen JF Korevaar DA Altman DG Bruns DE Gatsonis CA Hooft L . STARD 2015 guidelines for reporting diagnostic accuracy studies: explanation and elaboration. BMJ Open. (2016) 6:e012799. doi: 10.1136/bmjopen-2016-012799 PMC5128957 28137831

[13] Ahmed F Khan AA Ansari HR Haque A. A systems biology and LASSO-based approach to decipher the transcriptome-Interactome signature for predicting non-small cell lung Cancer. Biology (Basel). (2022) 11:1752. doi: 10.3390/biology11121752 36552262 PMC9774707

[14] Xu D Zhao X Ye D Huo C Peng X Liu Y . A gene-based predictive model for lymph node metastasis in cervical cancer: superior performance over imaging techniques. J Transl Med. (2025) 23:397. doi: 10.1186/s12967-025-06327-3, 40181462 PMC11969859

[15] Brenna SMF Zeferino LC Pinto GA Souza RA Andrade LAL Vassalo J . P53 expression as a predictor of recurrence in cervical squamous cell carcinoma. Int J Gynecol Cancer. (2002) 12:299–303. doi: 10.1136/ijgc-00009577-200205000-00010 12060452

[16] Aghajani Mir M Daraei A. Defective biological networks associated with pseudogene-derived lncRNAs in cancer drug resistance: promising prospects for their clinical targets in cancer therapy. Genes Dis. (2026) 13:101728. doi: 10.1016/j.gendis.2025.101728, 41362671 PMC12682027

[17] Hussein NH Amin NS El Tayebi HM. GPI-AP: Unraveling a new class of malignancy mediators and potential immunotherapy targets. Front Oncol. (2020) 10:10. doi: 10.3389/fonc.2020.537311, 33344222 PMC7746843

[18] Bray F Laversanne M Sung H Ferlay J Siegel RL Soerjomataram I . Global cancer statistics 2022: GLOBOCAN estimates of incidence and mortality worldwide for 36 cancers in 185 countries. CA Cancer J Clin. (2024) 74:229–63. doi: 10.3322/caac.21834, 38572751

