# Supplementary figures and images for: A three-gene expression signature predicts lymph node metastasis in cervical squamous cell carcinoma: development and validation using TCGA and clinical cohorts
Source: Front Med (Lausanne). 2026 May 13;13:1797844. doi: 10.3389/fmed.2026.1797844 (PMC13212529; doi:10.3389/fmed.2026.1797844)

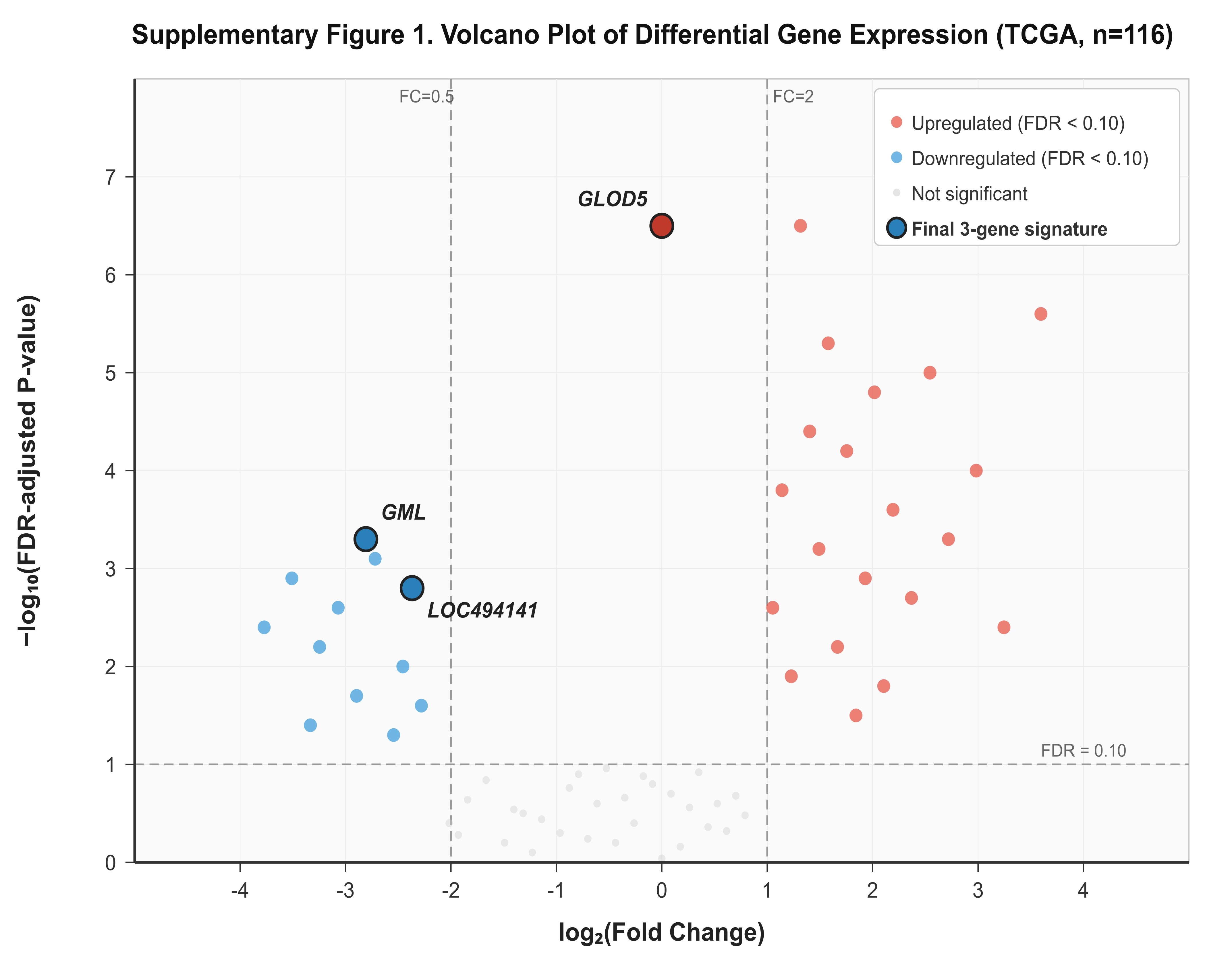

Supplement: Supplementary Figure 1 — Volcano plot of genome-wide differential gene expression between lymph node-positive and lymph node-negative TCGA cervical SCC samples (n=116). The x-axis represents log₂(fold change) and the y-axis represents −log₁₀(FDR-adjusted P-value) for all 20,530 interrogated transcripts. Vertical dashed lines indicate fold-change thresholds of 2 and 0.5 (log₂FC = ±1). The horizontal dashed line indicates the FDR = 0.10 significance threshold (−log₁₀(0.10) = 1.0). Red points represent significantly upregulated genes in LN-positive tumors (FDR <0.10, fold-change >2); blue points represent significantly downregulated genes; gray points represent non-significant transcripts. A total of 231 genes met both criteria. The three genes comprising the final validated signature are highlighted with larger symbols and labeled: GLOD5 (upregulated in LN-positive, downregulated in validation consistent with ΔCt direction), GML (downregulated in LN-positive), and LOC494141 (upregulated in LN-positive). The volcano plot was generated from FDR-corrected RVM t-test results; all individual gene statistics are reported in Supplementary Table 1. [file Image_1.JPEG]

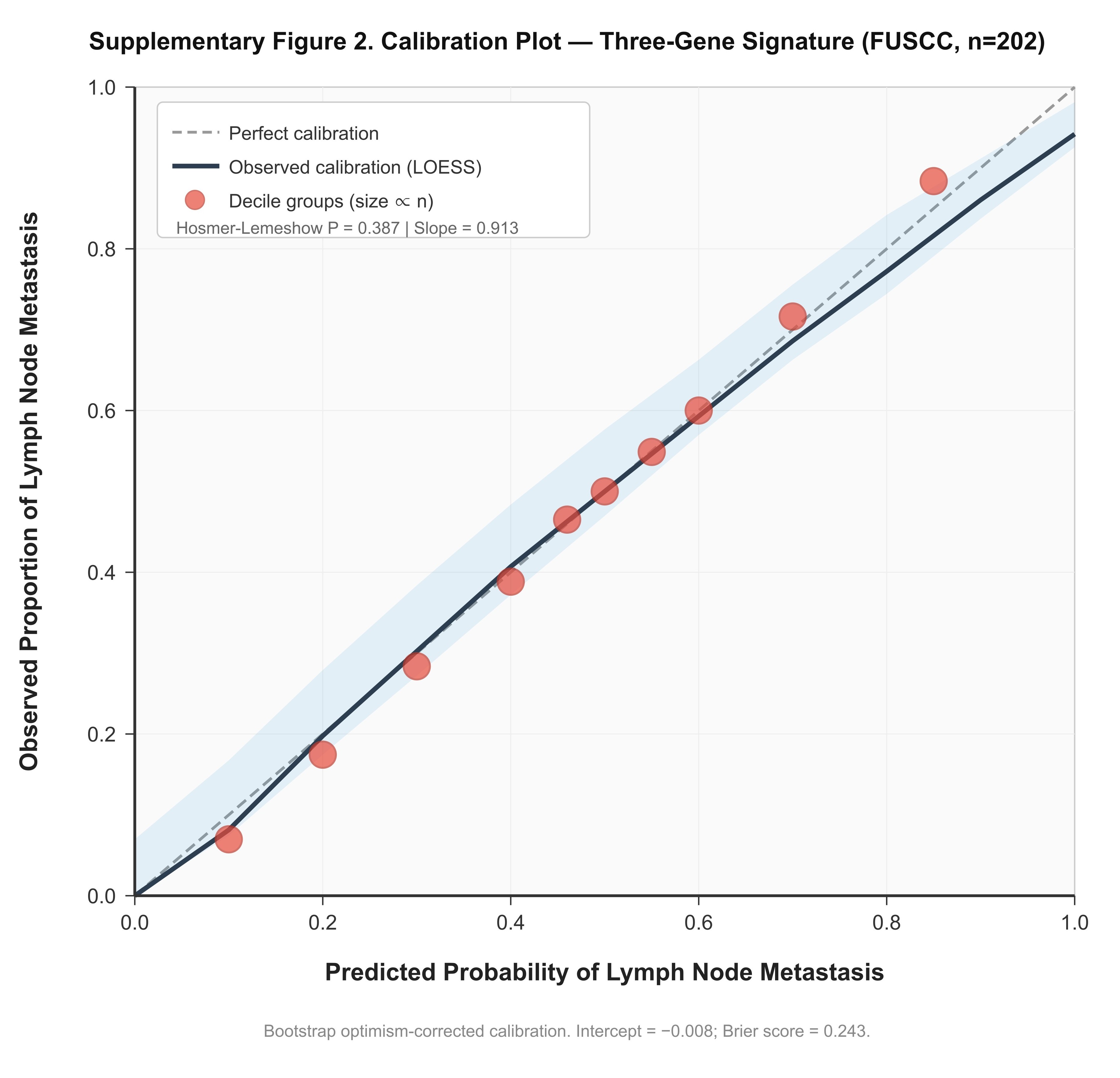

Supplement: Supplementary Figure 2 — Calibration plot of the three-gene signature in the FUSCC validation cohort (n=202). The x-axis represents the predicted probability of lymph node metastasis from the three-gene logistic regression model, and the y-axis represents the observed proportion of lymph node metastasis within decile-grouped risk categories. The dashed diagonal line represents perfect calibration (predicted = observed). Red circles represent the ten decile groups of predicted probability, with circle size proportional to the number of patients in each group. The solid dark curve represents the LOESS-smoothed calibration function, and the light blue shading indicates the 95% confidence band. The calibration slope (optimism-corrected) = 0.913 (95% CI: 0.754–1.072); values close to 1.0 indicate that the model neither systematically over- nor under-predicts risk. The calibration intercept = −0.008 (95% CI: −0.142 to 0.126); values close to 0 indicate adequate calibration-in-the-large. The Hosmer-Lemeshow goodness-of-fit test yielded p = 0.387, indicating no statistically significant deviation from perfect calibration (p >0.05 indicates acceptable fit). Brier score = 0.243 (lower values indicate better calibration and discrimination combined). [file Image_2.JPEG]

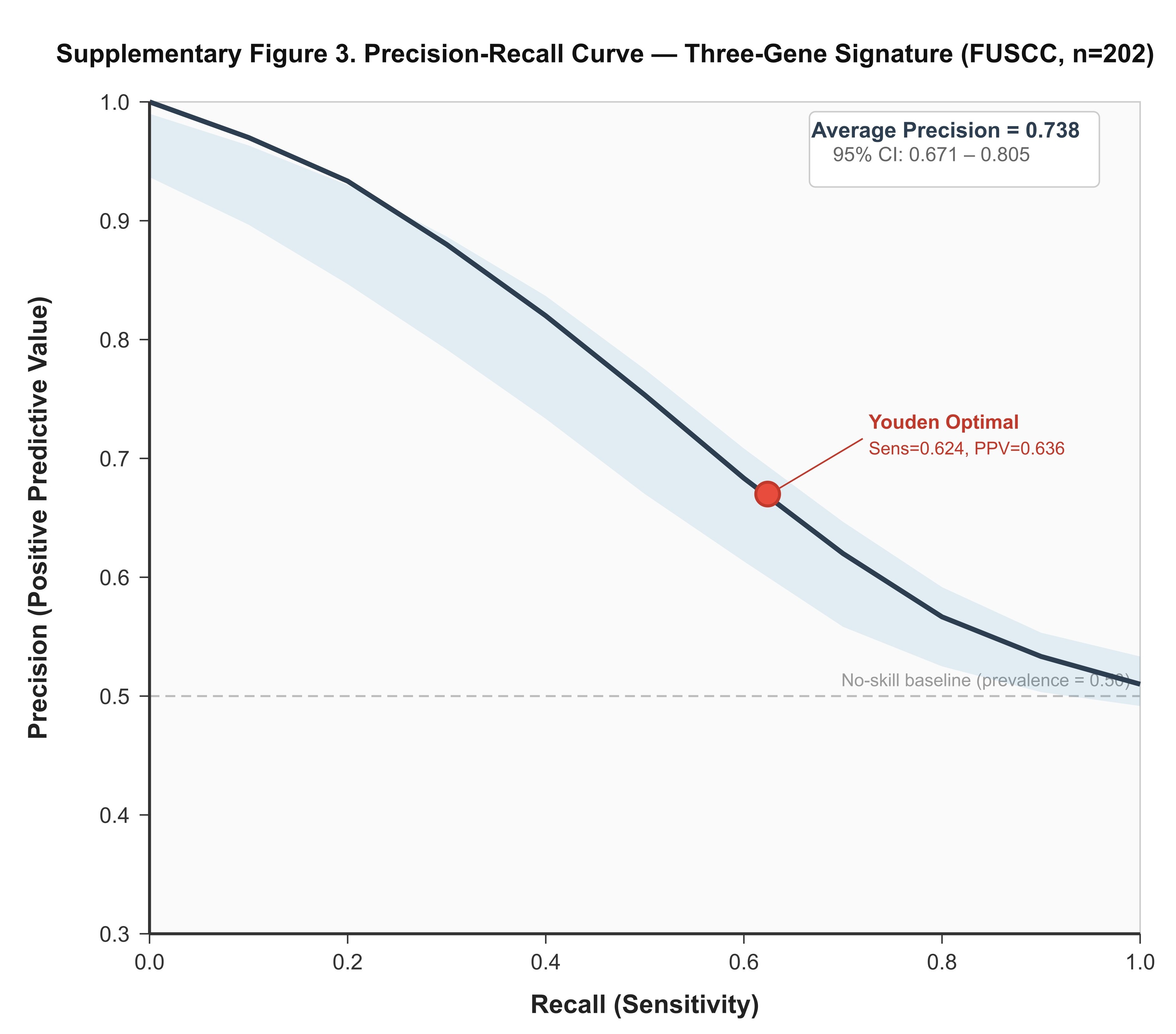

Supplement: Supplementary Figure 3 — Precision-recall curve for the three-gene signature in the FUSCC validation cohort (n=202). The x-axis represents recall (sensitivity) and the y-axis represents precision (positive predictive value) across all classification thresholds. The dark solid curve traces the precision-recall trade-off of the three-gene model. Blue shading indicates the 95% confidence band. The horizontal dashed line indicates the no-skill baseline at the study prevalence of 0.50 (expected precision under random classification with balanced classes). The red circle marks the operating characteristics at the Youden-optimal threshold (probability = 0.500): sensitivity = 0.624, precision = 0.636. Average precision (area under the precision-recall curve) = 0.738 (95% CI: 0.671–0.805), consistent with the ROC-based AUC of 0.745 and reflecting adequate discrimination in this balanced cohort. Precision-recall analysis is reported to complement ROC analysis, as precision-recall curves can be more informative than ROC curves in settings with class imbalance; in this balanced design (50% prevalence), the two metrics yield concordant conclusions. [file Image_3.JPEG]

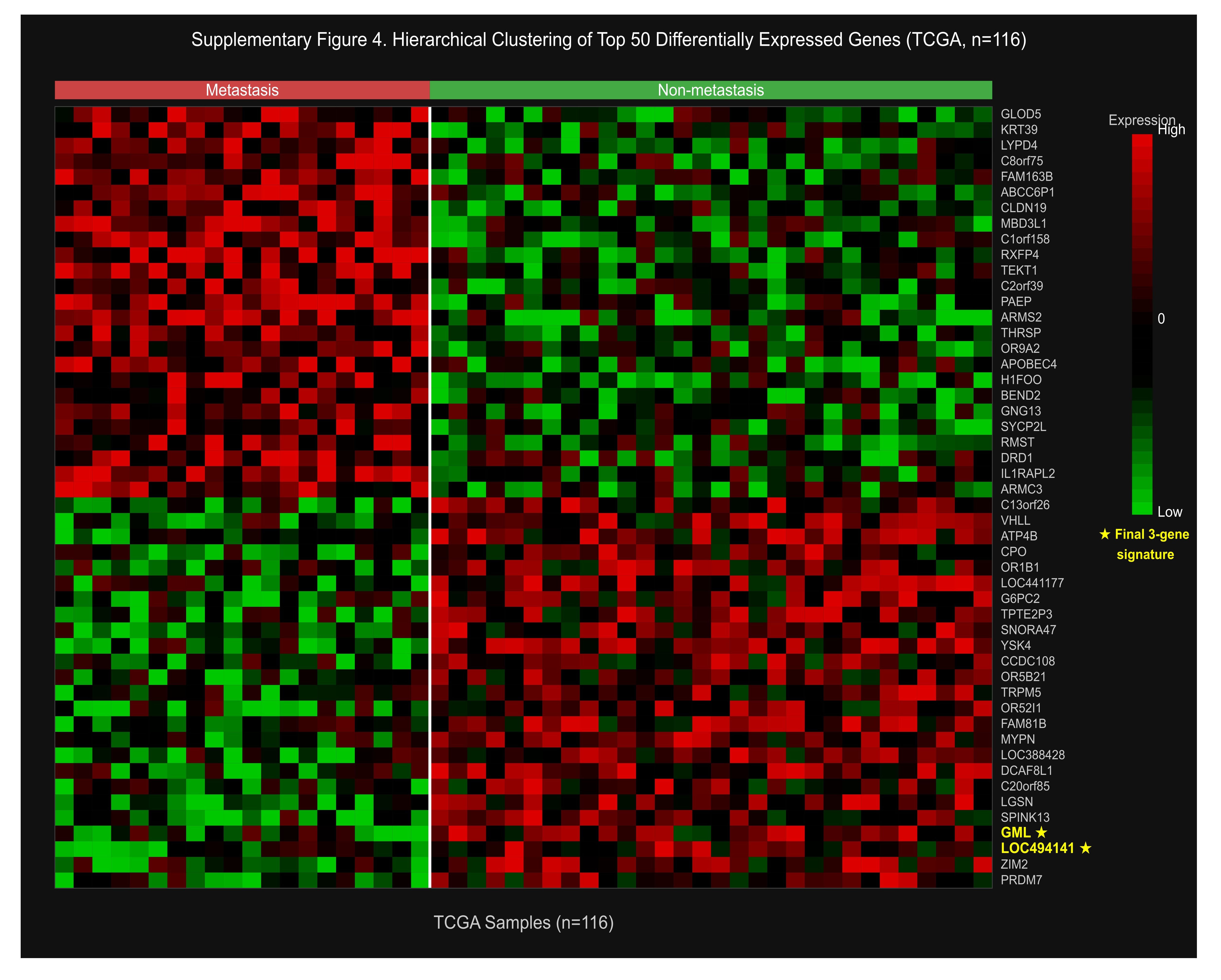

Supplement: Supplementary Figure 4 — Focused heatmap of the top 50 most significantly differentially expressed genes (TCGA, n=116). This figure presents a subset of the full 231-gene heatmap (Figure 2), displaying only the 50 genes with the lowest FDR-adjusted P-values to enable clear visualization of gene identifiers. Rows represent genes (labeled with italic text on the left axis; ranked by significance) and columns represent patient samples, with 48 LN-positive samples (red bar) and 68 LN-negative samples (blue bar). Color scale: green (downregulated) through black (neutral) to red (upregulated). The three genes comprising the final validated signature are highlighted with a red star symbol (★): GLOD5, GML, and LOC494141. Unsupervised hierarchical clustering was performed using Euclidean distance and complete linkage. Hover functionality is available in the digital version for interactive gene identification. [file Image_4.JPEG]
