# Supplementary material for: A three-gene expression signature predicts lymph node metastasis in cervical squamous cell carcinoma: development and validation using TCGA and clinical cohorts
Source: Front Med (Lausanne). 2026 May 13;13:1797844. doi: 10.3389/fmed.2026.1797844 (PMC13212529; doi:10.3389/fmed.2026.1797844)
Supplement: Supplementary file 6 [file Data_Sheet_2.docx]

**SUPPLEMENTARY MATERIALS**

**Supplementary Table 1. 231 mRNAs that were differentially expressed between LN-positive and LN-negative patients based on TCGA mRNA-seq database**

|  | foldchange | pvalue |  |  | foldchange | pvalue |
| --- | --- | --- | --- | --- | --- | --- |
| **GLOD5** | 2.107230593 | 3.11E-07 |  | **NPY** | Inf | 0.015521613 |
| **KRT39** | 2.548489504 | 2.73E-05 |  | **DDX3Y** | 3.379215079 | 0.01581917 |
| **LYPD4** | 10.01788186 | 8.98E-05 |  | **LOC653544** | Inf | 0.016304667 |
| **C8orf75** | 2.354080077 | 0.000123293 |  | **MAGEB4** | Inf | 0.01637462 |
| **FAM163B** | 3.003989754 | 0.000140916 |  | **KLK3** | 4.347536427 | 0.016607442 |
| **ABCC6P1** | 3.313877686 | 0.000142521 |  | **SCARNA4** | Inf | 0.016880559 |
| **CLDN19** | 3.319524896 | 0.00019457 |  | **TM4SF20** | 2.813182657 | 0.017023575 |
| **MBD3L1** | Inf | 0.000226598 |  | **LECT1** | 8.579326946 | 0.017473588 |
| **C1orf158** | 31.45590075 | 0.000235759 |  | **CPXCR1** | 3.253955046 | 0.017673876 |
| **RXFP4** | 2.220334831 | 0.000389964 |  | **LOC494141** | 0.355991645 | 0.018195036 |
| **TEKT1** | 8.771185527 | 0.000408588 |  | **LRRTM3** | 4.27153876 | 0.018255066 |
| **C2orf39** | 2.442877705 | 0.000452292 |  | **ESX1** | 9.099623048 | 0.018513855 |
| **CLEC4GP1** | 2.472978621 | 0.000459611 |  | **CA10** | 2.021510774 | 0.018690705 |
| **PAEP** | 2.508530508 | 0.000526023 |  | **OR52N2** | 4.120720166 | 0.019024691 |
| **ARMS2** | 3.059736452 | 0.000529349 |  | **CGA** | 2.893889937 | 0.019099417 |
| **THRSP** | 2.57685764 | 0.000545169 |  | **C6orf118** | 2.32473146 | 0.019297032 |
| **OR9A2** | Inf | 0.000566643 |  | **KCNH5** | 2.63900252 | 0.019321371 |
| **APOBEC4** | 4.037881328 | 0.000608634 |  | **HSD3B2** | 2.301870858 | 0.01939651 |
| **H1FOO** | Inf | 0.000609455 |  | **RPS4Y1** | 4.968033779 | 0.019714332 |
| **BEND2** | 8.998644704 | 0.000650592 |  | **OR1F2P** | 2.75855607 | 0.01990972 |
| **GNG13** | 2.732158747 | 0.000755193 |  | **KNCN** | 9.315410792 | 0.020164609 |
| **SYCP2L** | 2.216699701 | 0.000902241 |  | **HMHB1** | 3.734775159 | 0.020905469 |
| **RMST** | 16.27158048 | 0.001341001 |  | **GIP** | 5.744550701 | 0.021106273 |
| **DRD1** | 2.196692574 | 0.001443084 |  | **C15orf32** | Inf | 0.021147785 |
| **IL1RAPL2** | 2.882464928 | 0.00150557 |  | **NAP1L6** | 2.381967517 | 0.021166681 |
| **ARMC3** | 2.590050275 | 0.00151904 |  | **POU3F3** | 10.60080096 | 0.021407524 |
| **C13orf26** | 7.867563478 | 0.001590937 |  | **TMEM196** | 6.190508021 | 0.021457055 |
| **VHLL** | 8.403044204 | 0.001739991 |  | **MYT1L** | 2.987775393 | 0.021578308 |
| **ATP4B** | 2.755442663 | 0.001814704 |  | **SLC26A3** | 3.433764979 | 0.022154808 |
| **CPO** | 2.282631521 | 0.001895607 |  | **OR5E1P** | 3.618982803 | 0.022278621 |
| **OR1B1** | Inf | 0.001928088 |  | **C2orf73** | 2.112767621 | 0.02306988 |
| **LOC441177** | 4.531868738 | 0.002083837 |  | **NRAP** | 4.192174828 | 0.023148771 |
| **G6PC2** | Inf | 0.002213597 |  | **NHEG1** | 6.81415639 | 0.02332574 |
| **TPTE2P3** | 2.784102423 | 0.002408963 |  | **LOC100133469** | 7.629103882 | 0.023809707 |
| **SNORA47** | Inf | 0.002431112 |  | **OR52W1** | Inf | 0.024946013 |
| **YSK4** | 2.704431372 | 0.002657017 |  | **XAGE2** | 26.33256881 | 0.024959427 |
| **CCDC108** | 2.325920876 | 0.002996433 |  | **MYH8** | 5.223925111 | 0.025354033 |
| **OR5B21** | Inf | 0.003085991 |  | **SNORA56** | Inf | 0.025435899 |
| **TRPM5** | 2.143972776 | 0.003253934 |  | **WDR69** | 2.022277371 | 0.025550987 |
| **OR52I1** | Inf | 0.003406599 |  | **TRYX3** | Inf | 0.025605787 |
| **FAM81B** | 2.464305381 | 0.003487313 |  | **TTTY15** | Inf | 0.025617916 |
| **MYPN** | 2.282327231 | 0.003497772 |  | **SNORD15A** | 3.577390007 | 0.025788773 |
| **LOC388428** | Inf | 0.00374388 |  | **SNORA2A** | Inf | 0.026376011 |
| **DCAF8L1** | Inf | 0.003774162 |  | **KBTBD13** | 4.334066063 | 0.026494063 |
| **C20orf85** | 5.459878488 | 0.004070324 |  | **CRYBA1** | 4.078301261 | 0.026764121 |
| **LGSN** | 2.911691398 | 0.004121808 |  | **LOC100190940** | 3.166328293 | 0.026775094 |
| **SPINK13** | 3.819748837 | 0.004172727 |  | **CAPSL** | 2.060699654 | 0.026840667 |
| **C6orf176** | 2.086401048 | 0.004216701 |  | **PCA3** | 18.7342862 | 0.027134673 |
| **ARHGEF38** | 2.246036324 | 0.00423098 |  | **C7orf52** | 2.209421878 | 0.027674185 |
| **SCGB3A2** | 8.241406757 | 0.00424409 |  | **KRTAP5-11** | Inf | 0.027733216 |
| **LOC84931** | 4.606228349 | 0.004375809 |  | **DMRTC2** | 5.509230412 | 0.027738261 |
| **SHH** | 2.561222967 | 0.004696007 |  | **ZDHHC22** | 2.065347679 | 0.028170168 |
| **OR2L2** | Inf | 0.005093837 |  | **C4orf22** | 2.97412018 | 0.028380822 |
| **C22orf33** | Inf | 0.005111393 |  | **TRPM3** | 2.035482362 | 0.028407886 |
| **SH2D6** | 2.62885015 | 0.005278364 |  | **LOC285735** | 3.305395635 | 0.028835113 |
| **OR6B3** | Inf | 0.005459639 |  | **SCGB1D1** | Inf | 0.029101803 |
| **FAM163A** | 2.139142738 | 0.00546034 |  | **KRTAP4-11** | 13.17517741 | 0.029497989 |
| **FKSG83** | Inf | 0.005795659 |  | **LRRTM1** | 2.142090845 | 0.029645753 |
| **GUCY2GP** | Inf | 0.005998826 |  | **LOC348021** | Inf | 0.029716726 |
| **FAM9B** | 5.883691483 | 0.006243593 |  | **DBX2** | 0.41438317 | 0.030082713 |
| **DCDC2B** | 2.158694948 | 0.006305739 |  | **ZIM2** | 0.238496784 | 0.031210388 |
| **OR2AG2** | 6.779708818 | 0.006473057 |  | **MAB21L2** | 2.114394907 | 0.031232628 |
| **TMEM211** | 2.32318755 | 0.006714241 |  | **HDGFL1** | 5.55442035 | 0.032059897 |
| **ZBBX** | 2.826131304 | 0.006796728 |  | **NEUROD4** | Inf | 0.032358934 |
| **CXorf41** | 2.138880794 | 0.006808326 |  | **IQCJ** | Inf | 0.032614852 |
| **LCN1** | 11.75335672 | 0.00708457 |  | **11-Mar** | 9.824152317 | 0.032812636 |
| **SNTG2** | 2.272324766 | 0.007226361 |  | **C11orf34** | 2.483759765 | 0.033820201 |
| **CEACAM16** | 3.878751039 | 0.007445441 |  | **FXYD4** | 3.084276665 | 0.033912912 |
| **LAMB4** | 2.048575671 | 0.007616355 |  | **MYL10** | 4.048513666 | 0.034298821 |
| **KCTD4** | 2.298022644 | 0.00762752 |  | **KRTAP7-1** | Inf | 0.034440499 |
| **TAS2R1** | 10.19526689 | 0.008103467 |  | **ADAM18** | Inf | 0.034726236 |
| **ODF1** | Inf | 0.008294693 |  | **NOBOX** | 8.114072362 | 0.034915868 |
| **ATOH1** | Inf | 0.008342348 |  | **LOC100240726** | 2.173034325 | 0.0353911 |
| **RBP2** | 3.775197763 | 0.008425793 |  | **GPR112** | 11.06920557 | 0.035490354 |
| **TMEM229A** | 7.033476634 | 0.00847194 |  | **LIPM** | 4.921115683 | 0.035501999 |
| **FAM48B1** | 5.490595611 | 0.00868692 |  | **MYH4** | 0.187863855 | 0.036066877 |
| **TMEM179** | Inf | 0.008840668 |  | **SI** | Inf | 0.03607502 |
| **SPINLW1** | 2.123639488 | 0.009021775 |  | **IFLTD1** | 4.088072826 | 0.036398142 |
| **C20orf70** | 3.370110037 | 0.009083487 |  | **CCDC129** | 2.352583196 | 0.036537447 |
| **NXNL1** | Inf | 0.009198667 |  | **HRG** | 0.474645737 | 0.036671646 |
| **POTEG** | 9.934528774 | 0.009411928 |  | **C9orf4** | 3.120420064 | 0.037039789 |
| **SLC7A10** | 2.319751263 | 0.009552322 |  | **TFF2** | 2.111802415 | 0.037164738 |
| **WFDC6** | 7.441819619 | 0.00964053 |  | **UBQLN3** | Inf | 0.038623958 |
| **PABPC1L2B** | Inf | 0.009665216 |  | **C17orf73** | 5.09098227 | 0.039741755 |
| **CCDC37** | 2.636938337 | 0.009687859 |  | **CACNG5** | 3.557390885 | 0.039787736 |
| **RLBP1** | 3.873192314 | 0.009687914 |  | **SLC6A19** | 4.204409458 | 0.040464576 |
| **OSTBETA** | 2.478376492 | 0.009819155 |  | **KIR3DP1** | 11.10540132 | 0.040775907 |
| **GPR12** | 3.792769124 | 0.010059784 |  | **FGF20** | 8.606763473 | 0.040862354 |
| **C9orf171** | 2.114537774 | 0.010405409 |  | **CA5A** | 3.683973163 | 0.040884743 |
| **SPINK4** | 2.888561362 | 0.010872761 |  | **C20orf186** | 2.200872181 | 0.041049924 |
| **SNORA71D** | 5.188575297 | 0.010929731 |  | **SLC25A31** | 2.643302889 | 0.041130786 |
| **CYLC2** | Inf | 0.011253271 |  | **CA1** | 2.412947121 | 0.04122432 |
| **CA6** | 2.206255708 | 0.011702182 |  | **SHOX** | 3.522298662 | 0.041443303 |
| **TTLL8** | Inf | 0.012449474 |  | **FAM26D** | 3.711606681 | 0.041731932 |
| **LOC340357** | Inf | 0.012875185 |  | **LOC153910** | 2.259991003 | 0.042297142 |
| **PRDM7** | 0.424231023 | 0.013289771 |  | **NT5C1A** | 2.982426532 | 0.042954796 |
| **HSD3B1** | 11.04577965 | 0.013292658 |  | **GABRG1** | 5.199361777 | 0.042966577 |
| **PF4** | 2.435326171 | 0.013511506 |  | **LOC647309** | 2.82487238 | 0.043541096 |
| **C20orf166** | 4.934652816 | 0.013527183 |  | **PPP1R3A** | Inf | 0.043762721 |
| **MMP26** | Inf | 0.013686022 |  | **SPATA21** | 4.385818268 | 0.044487638 |
| **C11orf36** | 4.718053529 | 0.013771573 |  | **SNORA46** | 4.519136193 | 0.044594316 |
| **FLJ40504** | 2.798003866 | 0.013795708 |  | **HBG2** | 2.022879898 | 0.045328259 |
| **SNORA65** | 3.058217365 | 0.014119202 |  | **OR2B11** | 0.282131506 | 0.045989467 |
| **GML** | 0.277450287 | 0.014167229 |  | **IFNA2** | Inf | 0.045996178 |
| **LRRC67** | 4.212556054 | 0.014567345 |  | **SCGB1D4** | Inf | 0.046310536 |
| **ALPI** | 5.794966564 | 0.014874595 |  | **CALCA** | 5.901403023 | 0.046998437 |
| **C9orf135** | 3.172787377 | 0.014931111 |  | **MAGEB10** | Inf | 0.047101856 |
| **C12orf40** | 6.694367618 | 0.014944632 |  | **SCARNA3** | Inf | 0.047386184 |
| **GDEP** | 10.50573301 | 0.014958914 |  | **SGCZ** | 5.356535751 | 0.047581925 |
| **MRGPRE** | 3.032751587 | 0.015024961 |  | **SNORA42** | Inf | 0.047605066 |
| **NCRNA00113** | Inf | 0.015146598 |  | **IL31** | Inf | 0.048301503 |
| **PPIAL4E** | Inf | 0.015161363 |  | **RBM46** | 2.448006305 | 0.04956619 |
| **LHCGR** | Inf | 0.015197746 |  | **METTL11B** | 2.455899761 | 0.049747838 |
| **C14orf183** | 3.163660461 | 0.015379354 |  | **MAGEB1** | Inf | 0.04986968 |
| **C8orf74** | 3.257570377 | 0.015397315 |  | **ENAM** | 3.600676667 | 0.049954386 |

**Supplementary Table 2. Primer Sequences and Amplification Efficiencies for Seven Candidate Genes**

| **Gene** | **Primer** | **Sequence (5′→3′)** | **Product Size (bp)** | **Tm (°C)** | **Amplification Efficiency (%)** |
| --- | --- | --- | --- | --- | --- |
| APOBEC4 | Forward | GGGCTCCTGTTGTTTTTGTGC | 142 | 60.1 | 97.3 |
|  | Reverse | TGCCTTACGATATTCCTGGGT |  | 59.8 |  |
| LOC494141 | Forward | CTCCATTGCTTGCTTCTCTG | 168 | 59.5 | 94.8 |
|  | Reverse | TTCTTCTCTGTTCTGCCTCTT |  | 59.2 |  |
| MYH4 | Forward | CCAGTACAAATTCGGTCATACCA | 155 | 60.3 | 103.2 |
|  | Reverse | AGCTTTTCATCTCGCATTTCCT |  | 60.0 |  |
| GML | Forward | GACTTACAGTTTGAGATGCCA | 131 | 58.9 | 101.6 |
|  | Reverse | CGCCTAATATGATACGGACA |  | 58.4 |  |
| GLOD5 | Forward | AACAGTCATGGAGGGACAGC | 148 | 60.5 | 98.4 |
|  | Reverse | CATGGTGGTGTCTTTGATGC |  | 59.7 |  |
| HRG | Forward | CGGAGGCTGAGAAAGCTCTA | 139 | 60.2 | 92.1 |
|  | Reverse | ACTCTGTCCAAGTGGGCATC |  | 60.1 |  |
| ZIM2 | Forward | GGATGTGCTTGTGGACTTCA | 157 | 59.8 | 107.5 |
|  | Reverse | CAGGTTCCGGTAATTCTCCA |  | 59.3 |  |
| ACTB (Reference) | Forward | CATGTACGTTGCTATCCAGGC | 250 | 60.4 | 99.8 |
|  | Reverse | CTCCTTAATGTCACGCACGAT |  | 60.1 |  |

*All primers designed to span exon-exon junctions. Specificity confirmed by BLAST analysis and melt curve verification. Amplification efficiencies determined by serial dilution analysis (5-point, 10-fold dilutions). Acceptable range: 90–110%. The reference gene ACTB demonstrated a geNorm M-value of 0.38 (threshold <0.50).*

**Supplementary Table 3. Confusion Matrix for the Three-Gene Signature in the FUSCC Validation Cohort (n=202)**

|  | **Histopathology LN+** | **Histopathology LN−** | **Total** |
| --- | --- | --- | --- |
| **Predicted LN+** | 63 (True Positive) | 36 (False Positive) | 99 |
| **Predicted LN−** | 38 (False Negative) | 65 (True Negative) | 103 |
| **Total** | 101 | 101 | 202 |

**Derived Performance Metrics:**

| **Metric** | **Value** | **95% CI** |
| --- | --- | --- |
| Sensitivity (TP/[TP+FN]) | 62.38% | 52.4–71.5% |
| Specificity (TN/[TN+FP]) | 64.36% | 54.4–73.4% |
| PPV (TP/[TP+FP]) | 63.64% | 53.6–72.8% |
| NPV (TN/[TN+FN]) | 63.11% | 53.1–72.2% |
| Accuracy ([TP+TN]/Total) | 63.37% | 56.4–69.9% |
| Positive Likelihood Ratio | 1.75 | 1.32–2.32 |
| Negative Likelihood Ratio | 0.58 | 0.45–0.76 |
| Diagnostic Odds Ratio | 3.00 | 1.71–5.27 |

*Optimal cutoff probability = 0.500 (Youden index). Classification rule: LN+ if P(Y=1|X) > 0.500.* *The 38 false negatives represent the patients who would be misclassified as node-negative, underscoring the necessity for integration with complementary clinical and imaging assessments.*

**Supplementary Table 4. Threshold-Dependent Performance Analysis of the Three-Gene Signature**

| **Threshold Probability** | **Sensitivity (%)** | **Specificity (%)** | **PPV (%)** | **NPV (%)** | **+LR** | **−LR** | **Accuracy (%)** | **Correctly Classified** |
| --- | --- | --- | --- | --- | --- | --- | --- | --- |
| 0.30 | 85.15 | 33.66 | 56.21 | 69.39 | 1.28 | 0.44 | 59.41 | 120/202 |
| 0.35 | 78.22 | 47.52 | 59.85 | 68.57 | 1.49 | 0.46 | 62.87 | 127/202 |
| 0.40 | 72.28 | 54.46 | 61.34 | 66.27 | 1.59 | 0.51 | 63.37 | 128/202 |
| 0.45 | 67.33 | 59.41 | 62.39 | 64.52 | 1.66 | 0.55 | 63.37 | 128/202 |
| **0.50** | **62.38** | **64.36** | **63.64** | **63.11** | **1.75** | **0.58** | **63.37** | **128/202** |
| 0.55 | 54.46 | 72.28 | 66.27 | 61.34 | 1.96 | 0.63 | 63.37 | 128/202 |
| 0.60 | 45.54 | 79.21 | 68.66 | 59.26 | 2.19 | 0.69 | 62.38 | 126/202 |

*Bold row indicates the Youden-optimal threshold (P=0.500). In a screening context where sensitivity is prioritized to avoid missing metastatic disease, a threshold of 0.35 (sensitivity 78.2%, specificity 47.5%) may be clinically preferable. +LR: Positive likelihood ratio; −LR: Negative likelihood ratio. All confidence intervals available upon request.*

**Clinical interpretation:** At the lower threshold of 0.35, only approximately 22% of truly node-positive patients would be missed (false negative rate), compared with 38% at the standard 0.500 threshold. This gain in sensitivity comes at the cost of increased false positive referrals (52.5% of node-negative patients), which would necessitate additional imaging or surgical staging but would not result in direct patient harm.

**Supplementary Table 5. Prevalence-Adjusted Positive and Negative Predictive Values**

| **Assumed LNM Prevalence** | **PPV (%)** | **NPV (%)** | **Pre-test Probability LN+** | **Post-test Probability if Positive** | **Post-test Probability if Negative** |
| --- | --- | --- | --- | --- | --- |
| 15% | 23.6 | 90.7 | 15.0% | 23.6% | 9.3% |
| 20% | 30.4 | 87.3 | 20.0% | 30.4% | 12.7% |
| 25% | 36.8 | 83.7 | 25.0% | 36.8% | 16.3% |
| **50% (Study design)** | **63.6** | **63.1** | **50.0%** | **63.6%** | **36.9%** |

*Calculated using Bayes' theorem from the observed sensitivity (62.38%) and specificity (64.36%) of the three-gene signature at the 0.500 probability threshold. The true prevalence of lymph node metastasis in early-stage cervical SCC ranges from approximately 15–25% depending on FIGO stage distribution and institutional referral patterns.*

*Formulas applied:* *PPV = (Sensitivity × Prevalence) / [(Sensitivity × Prevalence) + ((1−Specificity) × (1−Prevalence))]* *NPV = (Specificity × (1−Prevalence)) / [((1−Sensitivity) × Prevalence) + (Specificity × (1−Prevalence))]*

**Clinical interpretation:** At realistic population prevalences (15–25%), the signature's NPV ranges from 84% to 91%, indicating that a negative molecular result provides meaningful reassurance regarding the absence of nodal metastasis. Conversely, the relatively low PPV (24–37%) at these prevalences indicates that a positive molecular result should prompt further confirmatory evaluation (enhanced imaging, sentinel lymph node biopsy) rather than immediate treatment escalation. This performance profile supports the signature's potential role as a "rule-out" tool within a tiered diagnostic algorithm.

**Supplementary Table 6. Subgroup Analysis of Three-Gene Signature Performance**

| **Subgroup** | **n (LN+/LN−)** | **AUC** | **95% CI** | **Interaction P-value** |
| --- | --- | --- | --- | --- |
| **Overall** | **202 (101/101)** | **0.745** | **0.676–0.814** | **—** |
|  |  |  |  |  |
| **FIGO Stage** |  |  |  | 0.82 |
| IB | 68 (31/37) | 0.731 | 0.612–0.850 |  |
| IIA | 83 (42/41) | 0.752 | 0.649–0.855 |  |
| IIB | 51 (28/23) | 0.739 | 0.601–0.877 |  |
|  |  |  |  |  |
| **Tumor Size** |  |  |  | 0.68 |
| <4 cm | 107 (48/59) | 0.738 | 0.645–0.831 |  |
| ≥4 cm | 95 (53/42) | 0.756 | 0.660–0.852 |  |
|  |  |  |  |  |
| **LVSI Status** |  |  |  | 0.41 |
| Positive | 113 (68/45) | 0.728 | 0.636–0.820 |  |
| Negative | 89 (33/56) | 0.761 | 0.660–0.862 |  |
|  |  |  |  |  |
| **Age Group** |  |  |  | 0.73 |
| <50 years | 110 (52/58) | 0.751 | 0.662–0.840 |  |
| ≥50 years | 92 (49/43) | 0.736 | 0.636–0.836 |  |

*AUC: Area under the ROC curve. Interaction P-values were calculated by adding an interaction term between the three-gene signature score and each clinical variable in the logistic regression model. All interaction P-values >0.20, indicating consistent signature performance across subgroups. Confidence intervals calculated by DeLong's method. The relatively wide confidence intervals in smaller subgroups reflect reduced statistical power and should be interpreted cautiously.*
